# Immune and Immune‐Integrated Organoids as NextGeneration Platforms for Disease Modeling

**DOI:** 10.1002/mco2.70531

**Published:** 2025-12-18

**Authors:** Kimiya Rashidan, Malaksima Ayadilord, Ali Hazrati, Amirhossein Nazerian, Abbas Shafiee, Seyed Mahmoud Hashemi

**Affiliations:** ^1^ Department of Immunology, School of Medicine Shahid Beheshti University of Medical Sciences Tehran Iran; ^2^ Department of Immunology, School of Medicine Tehran University of Medical Sciences Tehran Iran; ^3^ School of Medicine Iran University of Medical Sciences Tehran Iran; ^4^ The University of Queensland Frazer Institute, Faculty of Health Medicine and Behavioral Sciences The University of Queensland Brisbane Queensland Australia

**Keywords:** cancer, immune cell, inflammation, organoid, pluripotent stem cell, regenerative medicine

## Abstract

Organoids are three‐dimensional structures that closely resemble the architecture and functions of human organs, offering key advantages over traditional models by better replicating tissue complexity and cellular interactions. These systems have become invaluable tools for disease modeling, drug screening, and regenerative medicine applications. Despite this progress, their lack of immune components limits their usefulness in diseases where immune cells are central drivers of pathology and therapy. The absence of an immune system within organoids limits their physiological relevance, particularly for cancer, inflammation, and autoimmunity research. Immune cell‐containing organoids provide a comprehensive platform for immunotherapy, host–pathogen interactions, regeneration, and immune disorders. This review first highlights the transformative potential of immune cell‐containing organoids across cancer, infection, inflammation, autoimmunity, regeneration, and the modeling of primary lymphoid organs. It then examines current strategies for integrating immune cells into organoids, the variety of immune cell sources employed, and the challenges in maintaining immune cell function. Finally, the role of bioengineering, biobanking, and artificial intelligence in overcoming existing limitations and enhancing immune system modeling is discussed. Overall, this study positions immune cell‐containing organoids as powerful platforms for translational research and precision medicine.

## Graphical abstract

This graphical abstract outlines the sources and methods for integrating immune cells into organoids. Immune cells can be derived from peripheral blood, tissues, or differentiated from human‐induced pluripotent stem cells. The immune cells are incorporated into the organoid by using coculture techniques, direct injection, as well as scaffolds and air–liquid interface systems. The immune‐competent organoids are used for disease modeling and immunological research.

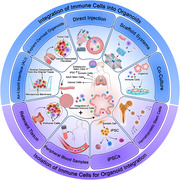

## 1 Introduction

**FIGURE 1:**
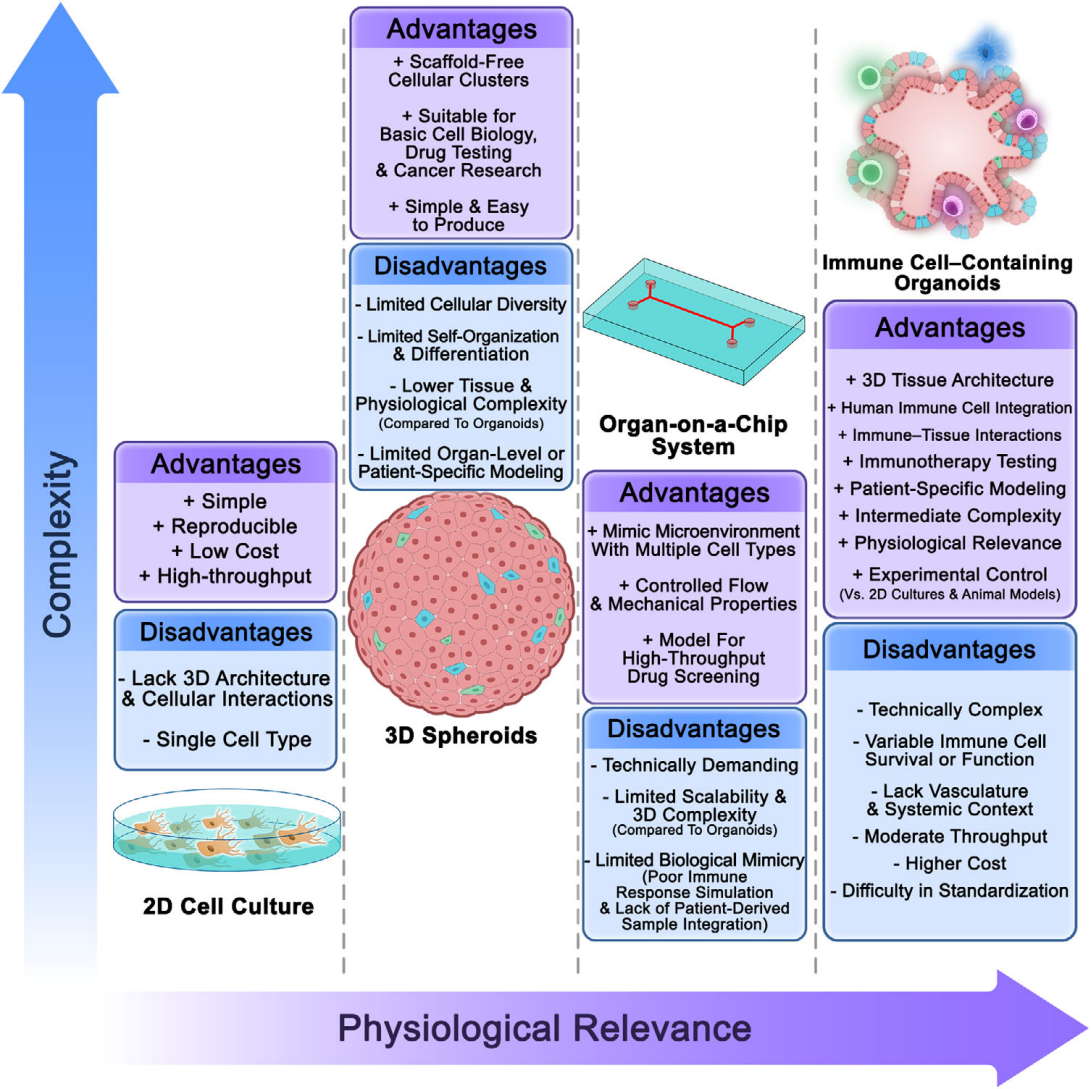
Comparison of immune cell‐containing organoids with other common disease modeling methods. Different platforms for disease modeling are summarized, highlighting their respective advantages and disadvantages.

Two‐dimensional (2D) cell cultures were developed in the early 20th century [1, 2, 3]. This was followed by the establishment of immortalized cell lines, such as HeLa cells in 1951, which allowed for long‐term cell studies but lacked the complexity of human tissues [4, 5, 6, 7]. The limitations of 2D cultures, particularly their inability to replicate the three‐dimensional (3D) structure and physiology of organs, led to the development of 3D cell cultures in the 1980s, where cells were grown in extracellular matrix (ECM)‐like environments [8, 9, 10]. The different platforms for disease modeling, along with their advantages and disadvantages, are summarized in Figure 1.

**FIGURE 2:**
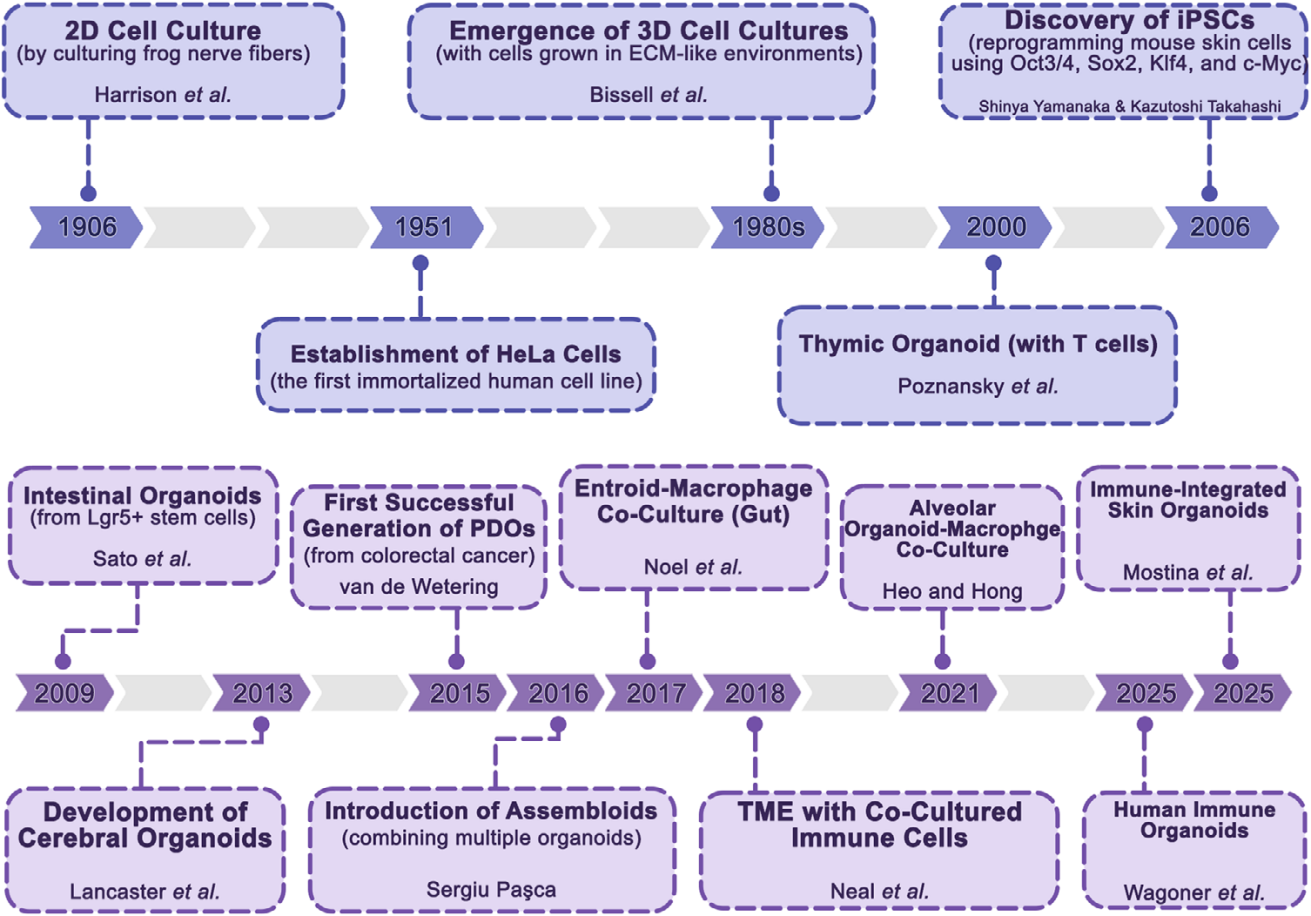
Historical milestones in organoid research from 2D cell culture to immune cell‐containing organoids.

The discovery of induced pluripotent stem cells (iPSCs) in 2006 was followed by reprogramming mouse skin cells into pluripotent stem cells using four key genes (Oct3/4, Sox2, Klf4, and c‐Myc). This discovery revolutionized regenerative medicine and disease modeling, enabling the generation of patient‐specific cells [1, 2, 3]. The first organoids emerged in 2009, with the creation of intestinal organoids from Lgr5+ stem cells, demonstrating self‐organization and functionality similar to the small intestine [11]. This was followed by the development of cerebral organoids in 2013 by Madeline Lancaster and colleagues, who used iPSCs to create brain‐like structures with neuroepithelial outgrowths, marking a significant step forward in neuroscience research [12]. Consequently, semicomplete skin organoids with hair follicles were introduced by Lee et al. in 2020 [13], offering potential for studying skin development and serving as a strong platform for dermatology research, or skin graft applications. Generally, an organoid is a 3D cell culture created from stem cells or tissue samples that replicates the structure, composition, and functionality of the corresponding tissue [14, 15]. Organoids can be produced from iPSCs, embryonic stem cells (ESCs), and adult stem cells (ASCs) through a process comparable to the development of their specific organization [16]. Furthermore, human organoid models of gut [17], lungs [18], kidneys [19], heart [20], liver [21], skin [22], and blood vessels [23] are now being developed, serving as versatile tools for modeling infectious diseases, genetic disorders, inflammatory conditions, regenerative medicine, and vaccine development [24, 25, 26, 27, 28]. In parallel, efforts were made to create organoids comprising immune cells to build more relevant organs and tumor models. For example, in 2017, Noel et al. established an enteroid with cocultured macrophages to explore gut physiology and host–pathogen interactions [29]. In 2021, the first alveolar organoid comprising macrophages was successfully made by Heo and Hong to study pulmonary fibrosis [30] (Figure 2).

3D organoid cultures from patient tumor samples have been developed in the past decade. These cancer‐derived organoids are often grown in an ECM, such as Matrigel, and mimic the original tumor's characteristics [31]. Tumor organoids have advanced molecular cancer research by providing a more realistic representation of the tumor tissue to study tumor biology, molecular pathways, and therapeutic responses compared to the conventional 2D‐grown tumor cell lines [32, 33]. Due to their ability to replicate the genetic characteristics of the parental cancer, organoids are regarded as a promising paradigm in precision medicine [34]. To date, organoids have been established for the majority of solid cancers, and many studies are currently being conducted to investigate their potential for guiding patient treatment [35]. More specifically, patient‐derived organoids (PDOs) have emerged as a transformative platform for precision medicine. PDOs, first successfully generated from colorectal cancer in 2015, modeling the genetic, molecular, and histological characteristics of primary tumors, making them invaluable for drug screening and personalized treatment strategies [36]. Due to the importance of immune cells in the microenvironment of tumors and response to immunotherapies, PDOs with integrated immune cells were soon developed [35, 37]. The integration of PDOs with CRISPR‐Cas9 gene editing has further enhanced their utility, allowing researchers to introduce specific mutations and study their impact in a controlled environment [38, 39].

**FIGURE 3:**
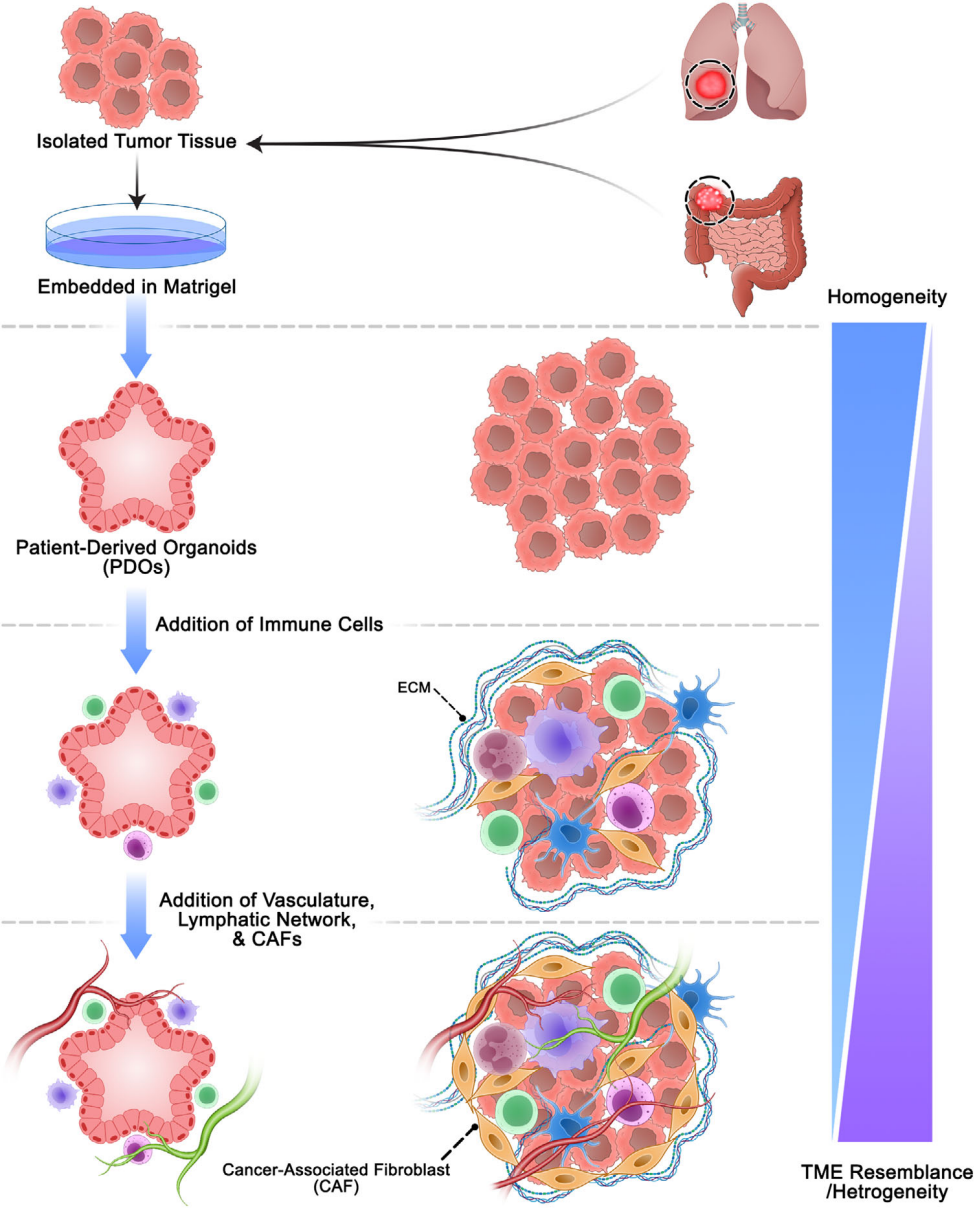
Advancing PDO complexity to resemble the TME. PDOs are first generated by embedding tumor tissue in Matrigel, and subsequent incorporation of immune cells, vasculature, lymphatic networks, and CAFs progressively enhances model complexity, enabling more accurate studies of cancer progression and immunotherapeutic responses. CAFs, cancer‐associated fibroblasts; PDO, patient‐derived organoids.

The complexity of organoids advanced further with the introduction of assembloids in 2016 [40]. Assembloids are more complex 3D structures formed by combining two or more distinct organoids, or by integrating other cell or tissue types into organoids [41]. These structures could be used to model interactions between different brain regions, such as the cortex and thalamus, providing a more sophisticated model for studying neural circuits and neurodevelopmental disorders [42, 43]. Recent innovations include the use of bioengineered tools like miniaturized bioreactors and microfluidic chips to overcome limitations such as vascularization and nutrient diffusion [44, 45]. These advancements have positioned organoids and assembloids as powerful tools for modeling human development and disease, bridging the gap between traditional 2D cultures and in vivo models [46, 47] (Figure 3). Despite these advancements, challenges remain, such as the lack of immune cells, vascularization, nerves, and microbiome in current organoid models, which limit their ability to fully replicate the organ or the tumor microenvironment (TME) [45, 46, 48, 49, 50, 51, 52]. Therefore, the inclusion of immune cells enhances the organoids' precision as a laboratory model, bringing them closer to mimicking the organism's physiological conditions [46, 53]. Given the growing importance of these systems, this review aims to provide a comprehensive overview of immune cell‐containing organoids, highlighting their development, applications, and future potential.

We summarize their role in modeling cancer, infection, inflammation, autoimmunity, tissue damage, and wound healing. We also discuss progress in constructing lymphoid organoids that mimic the bone marrow (BM), thymus, lymph nodes, and spleen. Furthermore, the review examines the sources of immune cells, methods for their incorporation, and the challenges of maintaining immune cell viability and function within organoid cultures. Finally, we discuss future directions, with particular emphasis on bioengineering, biobanking, and artificial intelligence (AI). Together, the current study outlines the development and applications of immune cell‐containing organoids, advancing disease pathogenesis research and hold promise for personalized therapies for currently incurable conditions.

## 2 Immune Cell‐Containing Organoid Models of Cancer and Immunotherapy

### 2.1 Key Immune Cells in Tumor Progression

Tumors comprise neoplastic and non‐neoplastic host components, collectively referred to as the TME. The TME has a complex structure and a distinctive immunological composition, which includes natural killer (NK) cells, macrophages, dendritic cells (DCs), lymphocytes, and myeloid‐derived suppressor cells (MDSCs) [54]. Understanding cell–cell interactions within a tumor is essential for improving treatments, as the TME influences cancer progression and drug response.

Tumor‐infiltrating lymphocytes (TILs) play a key role in anticancer immune responses. However, these lymphocytes can also lead to immunosuppression, which may facilitate tumor development [55]. Throughout cancer progression, anticancer macrophages initially exhibit M1‐like polarization, facilitating the elimination of immunogenic malignant cells. However, as tumors grow, the TME drives the M2‐like polarization of tumor‐associated macrophages (TAMs), promoting tumorigenesis [56]. TAMs release cytokines like IL‐10 [57] and TGF‐β [58], which mediate immunoregulation, limit effector T‐cell function, restrict DC maturation and promote tumor cell expansion [59]. Furthermore, NK cells serve vital roles in identifying and eliminating cancer cells and participate in the early immune responses to malignancies. Nevertheless, NK cells may be suppressed by immunoregulatory signals within the TME [60, 61]. Antigen presentation and T‐cell activation are critical functions of DCs. DC efficacy might be reduced in TME because of regulatory agents [61]. CD8+ cytotoxic T lymphocytes (CTLs) target and destroy cancerous cells. CD4+ T helper (Th1) cells generate cytokines such as IL‐2 and IFN‐γ, supporting the activity of CTLs [62]. Tregs and MDSCs assist in sustaining immunological tolerance and thereby can suppress anticancer reactions, resulting in an immunoregulatory state [63]. All of the mentioned immune cells play critical roles in cancer progression and incorporating them into organoids could enhance the physiological and translational relevance of these models.

### 2.2 Applications of Immune Cells‐Containing Organoids in Cancer Research

Due to the limited ability to predict each patient's response to treatment, some patients might receive poor care that exposes them needlessly to harmful side effects and incurs significant treatment expenses. To enable personalized treatment and improve cancer patient survival, effective predictive biomarkers are required [64].

In recent years, advancements in the understanding of tumor biological activities, particularly tumor heterogeneity, have led to personalized tumor treatment guided by molecular classification [65, 66, 67]. To delve deeper into the processes of cancer development, tumor invasion, and metastasis and how these might be applied to new treatment options, in vitro models are essential. A gap exists between the modeling systems and the in vivo conditions of patients, leading to the inaccuracy of the predictive capabilities of various preclinical models.

To bridge the gap, patient‐derived models, such as patient‐derived primary tumor cells (PDTCs), PDOs, and patient‐derived xenografts (PDXs), have been established as preclinical models for almost all types of solid tumors [68].

PDOs are 3D self‐organizing structures made of epithelial cells, produced from stem cells and have been developed for a range of tumors to mimic them in vitro. PDOs can be used to screen anticancer drugs ex vivo to predict clinical response [69, 70, 71]. Key advantages of PDOs over PDX are faster generation (days to weeks) and the ability to create normal tissue organoids for assessing off‐target effects [72]. Because of their inherent variability, long‐term stability, suitability for high‐throughput screens, and improved ability to show tumor features, PDOs are better preclinical models than their earlier models [36, 73, 74]. PDO biobanks of multiple cancer types, including colorectal [75, 76, 77], pancreatic [78, 79, 80], ovarian, and liver cancers [81], have been developed for conducting high‐throughput drug screening [36, 78, 82, 83, 84, 85, 86].

Nonetheless, the main limitation of PDO models is their absence of TME components, in particular immune cells, which play a crucial role in cancer progression and therapy response [37, 87]. To mimic cancer–stroma interactions, PDOs can be cocultured with stromal or immune cells just like recently established PDO platforms of various cancers, including bladder [88], pancreatic [37, 89], central nervous system (CNS) tumors [90], melanoma [91, 92], colorectal [37, 93, 94], renal cell carcinoma [37, 92], and non–small cell lung cancer (NSCLC) [37, 92] (Table 1). Zhou et al. developed a T‐cell‐incorporated pancreatic tumor organoid model for high‐throughput drug screening. Pancreatic tumor organoids of specific size and structure were produced from orthotopic pancreatic tumors in mice or from surgical tumor tissue samples of patients. This model replicated the cellular composition and immunosuppressive TME of the original tumor, with tumor‐specific T cells often localized on the organoid's periphery. Pancreatic tumor organoids comprising T cells served as an effective and functional model for investigating T‐cell infiltration, cytotoxicity, and interactions with tumor cells and their microenvironment [95].

**TABLE 1:** Development of immune cell‐containing organoids and their application in modeling cancer.

Cancer	Source of organoid	Immune cell	Source of immune cell	Integration method	Purpose	Year of publication	References
Bladder	Tumor tissue	T cell	Tissue biopsy	Coculture in Matrigel	Model regenerative tissue responses and replicate urothelial carcinoma pathology	2020	[88]
Pancreatic	Tumor tissue	T cell	PBMC	Coculture in Matrigel	Establish and characterize pancreatic cancer organoids and coculture models for disease study	2018	[89]
Pancreatic, colorectal, renal cell carcinoma, NSCLC	Tumor tissue	T cell, B cell, NK cell, macrophage	Tissue biopsy	ALI	Modeling responses to ICB therapy	2018	[37]
CNS	Tumor tissue	CD8‐positive lymphocyte	Tissue biopsy	Explant‐derived (already‐resident)	Modeling responses to ICB therapy	2019	[90]
Melanoma	Tumor tissue	Immune cells derived from lymph node tissue or PBMCs	Tissue biopsy and PBMC	Hydrogel biofabrication	Personalized immunotherapy screening	2020	[91]
Colorectal	Tumor tissue	T cell	Tissue biopsy	Coculture in Matrigel	Modeling responses to neoadjuvant/ICB t immunotherapy	2020	[93]
Colorectal	Tumor tissue	TIL	Tissue biopsy	Explant‐derived (already‐resident)	Modeling responses to ICB therapy	2018	[94]
Melanoma, NSCLC, BC, ovarian cancer, renal cell carcinoma	Tumor tissue	Tumor‐resident immune cells (T cell, B cell, NK cell)	Tissue biopsy	Explant‐derived (already‐resident)	Modeling responses to ICB therapy	2021	[92]
Pancreatic	Tumor tissue	T cell	Tissue biopsy	Coculture in Matrigel	Modeling responses to ICB therapy	2023	[95]
Melanoma	Tumor tissue	TIL	Tissue biopsy	Explant‐derived (already‐resident)	Modeling responses to ICB therapy	2021	[96]
CRC, NSCLC	Tumor tissue	PBL	PBMC	Coculture in murine basement membrane matrix (Geltrex)	Analyzing tumor‐specific T‐cell responses and isolating them	2018	[97]
CRC, NSCLC	Tumor tissue	PBL	PBMC	Coculture in murine basement membrane matrix (Geltrex)	Analyzing tumor‐specific T‐cell activity	2020	[98]
Pancreatic	Tumor tissue	T cell	PBMC	Coculture in Matrigel	Expanding and identifying tumor‐targeting T cells	2021	[99]
HGSC	Tumor tissue	Intratumoral immune cells (CD8⁺ T cells, CD4⁺ Tregs, NK cells, macrophages)	Tumor tissue	Coculture in Matrigel	Modeling responses to ICB therapy	2021	[100]
Melanoma and BC	Tumor tissue	T cell	PBMC	Acoustic virtual 3D scaffold (AV‐Scaf)	Modeling 3D tumor organoids and immune cells that interact directly	2024	[101]

Abbreviations: ALI, air–liquid interface; AV‐Scaf, acoustic virtual 3D scaffold; B cell, B lymphocyte; CD8, cluster of differentiation 8; CD8⁺, cluster of differentiation 8 positive; CD4⁺ tregs, cluster of differentiation 4 positive regulatory T cells; CRC, colorectal cancer; HGSC, high‐grade serous carcinoma; ICB, immune checkpoint blockade; NK cell, natural killer cell; NSCLC, non–small cell lung cancer; PBL, peripheral blood lymphocyte; PBMC, peripheral blood mononuclear cell; T cell, T lymphocyte; TIL, tumor‐infiltrating lymphocyte.

PDOs are also valuable platforms for evaluating the efficacy of immune checkpoint inhibitors (ICIs) by incorporating both tumor and immune cell compartments. Neal et al. established an air–liquid interface (ALI) coculturing system in which tumor organoids and immune cells were cultivated from minced primary tumor samples embedded in a collagen matrix [37]. The ALI platform preserved native epithelial, stromal, and immune cells, including T, B, NK cells, and macrophages. This design replicated the original TME by facilitating the proliferation of other cell types, including myofibroblasts and various immune cells. Nineteen human tumor–organoid cultures were generated, including kidney, pancreatic, and lung tumors. The study demonstrated that the TILs from the culture can maintain the T‐cell receptor (TCR) repertoire of the original tumor‐resident lymphocytes and can be stimulated by checkpoint inhibitors (anti‐PD1, anti‐PDL1) to augment antitumor cytotoxicity [37]. This method effectively models the TME, facilitating the modeling and monitoring of immune suppression and in vitro drug testing. The organoid‐based culture of primary tumor epithelium with endogenous immune stroma is expected to enhance immuno‐oncology research within the TME and support personalized immunotherapy evaluation [37].

When PDOs are combined with stromal and immune components, they are also referred to assembloids. For instance, PDOs from rectal cancer biopsies combined with autologous TILs identified all six patients achieving pathologic complete responses to neoadjuvant therapy [94]. Adding anti‐PD‐1 increased TIL cytokine production and cytotoxicity, confirming assembloids' responsiveness to immunotherapy. Similarly, melanoma assembloids incorporating lymph nodes or peripheral blood lymphocytes (PBLs) screened against treatments (e.g., nivolumab, ipilimumab) showed a correlation with clinical outcomes in 85% (6/7) of cases [91]. In a clinical trial study on neoadjuvant ICIs (ipilimumab plus nivolumab) in colon cancer, PDOs cocultured with PBL correlated with clinical outcomes in 75% (9/12) of cases based on CD8+ T cell IFN‐γ responses [93]. These findings highlight the potential of PDO‐based coculture systems as predictive platforms for investigating immunotherapy response. By effectively modeling tumor–immune interactions, these systems provide valuable insights into treatment efficacy and patient outcomes, paving the way for more personalized and effective cancer immunotherapies.

However, the success of PDO establishment differs across cancer subtypes [72], with nonepithelial cancers, apart from melanoma, being particularly difficult to model [84]. Furthermore, using peripheral immune components with tumor PDOs has limitations due to the absence of native tumor‐infiltrating immune cells, such as ICI‐responsive T cells [96].

Future efforts should focus on optimizing culture conditions, standardizing assays, and increasing sample sizes to fully unlock the potential of PDO‐based precision medicine. Additionally, AI algorithms could play a role in enhancing the design, construction, and functionality of immune–organoid models. These possibilities are further elaborated in the conclusion and future perspectives section.

The potential of immune cell‐containing organoids to model diseases extends beyond cancer. This platform can also be applied to conditions such as infection, inflammation, and autoimmunity, which will be discussed in the following sections.

## 3 Immune Cell‐Containing Organoid Models of Infection, Inflammation, and Autoimmunity

### 3.1 Key Immune Cells in Infection, Inflammation, and Autoimmunity

Inflammation is a major immune response to infection or injury, helping restore and maintain tissue homeostasis [102]. Localized immune cells modulate inflammation and promote tissue healing through resident cell functions and infiltrating regulatory immune cells [103]. These cells respond to microbial exposure in a quick and controlled manner, mediating immediate beneficial reactions, recruiting other immune cells to the sites of infection, and resolving the response to minimize potentially harmful consequences [104, 105]. Innate immunity against infections is based on immune cells' surface pattern recognition receptors (PRRs), which allow a small subset of immune cells to rapidly recognize and respond to a wide range of pathogens that have similar characteristics, or pathogen‐associated molecular patterns (PAMPs) [106]. Numerous immune cells are part of this response, including phagocytes (macrophages and neutrophils), DCs, mast cells, basophils, eosinophils, NK cells, and innate lymphoid cells [107]. These cells produce key inflammatory cytokines such as tumor necrosis factor (TNF), interleukin 1 (IL‐1), and interleukin 6 (IL‐6) in the early phases of the response to bacterial infection [108, 109]. These cytokines are critical due to their role in initiating local inflammation and recruiting immune cells. Macrophages trigger inflammation by releasing IL‐1β, IL‐6, IL‐8, CXCL1, CCL2, CCL7, and G‐CSF, which attract monocytes, neutrophils, NK cells, and effector T cells toward the infected site [110, 111]. Additionally, IL‐15 and IL‐18 stimulate NK cells [112]. TRM, gamma delta (γδ) T, and NK cells produce IFN‐γ, TNF‐α, IL‐2, perforin, and granzyme B. Activated CD4^+^ T cells support the recruitment of B cells to produce pathogen‐specific neutralizing antibodies [113, 114].

Inflammatory responses occur in both infection and autoimmune disorders. Infection is typically characterized by an acute, pathogen‐triggered recruitment of innate effectors (e.g., neutrophils, inflammatory macrophages), and pathogen‐specific adaptive responses (CD8^+^ T cells for viruses, Th1/Th17 cells for many bacteria/fungi) [115], whereas autoimmunity often features chronic, antigen‐specific adaptive responses with autoreactive CD4 T cells, B cells/plasma cells producing autoantibodies, and dysregulated regulatory T cells [116]. Although, both processes generate inflammatory responses, they differ in their time course, antigen specificity, and dominant effector mechanisms. Often, autoimmune or inflammatory diseases are linked to dysregulated cytokine production [117]. Whereas acute inflammation protects the body, chronic inflammation can injure tissues and disrupt their normal functioning [118]. Chronic inflammation can also be a major main mechanism underlying disease pathogenesis in various autoimmune diseases and contribute to disease progression. Local immune system reactions may develop situations like rheumatoid arthritis and systemic lupus erythematosus, resulting in the destruction of tissues caused by the release of autoantibodies and inflammatory agents, as well as improper stimulation of immune cells for tissue‐specific self‐antigens [119, 120]. A lack of balance among effector and modulatory cells could accelerate these illnesses, emphasizing the necessity of maintaining tissue‐specific immunological homeostasis as a treatment option [121]. Knowing how infections form and develop over time is necessary to fight them. Current research methods, including in vitro and animal models, fall short in accurately replicating and ultimately understanding human conditions [25, 122].

### 3.2 Applications of Immune Cells‐Containing Organoids in Modeling Infection, Inflammation, and Autoimmunity

Human stem cell‐derived organoid cultures are novel, promising models for studying infectious diseases [123, 124]. Respiratory infectious pathogens enter via the respiratory tree's lining, and airway organoid cultures are essential in studying these infections [125]. The organoids engineered from pluripotent or adult tissue stem cells include several cell types, such as basal cells, goblet cells, ciliated cells, club cells, and alveolar cells, and are able to recapitulate the respiratory epithelium [126]. They can also be cultured under conditions much closer to natural epithelium, such as ALI conditions [127]. In addition, lung organoids are only now being explored to investigate many infections caused by bacteria, parasites, fungi, and viruses, including parainfluenza virus 3, influenza, enterovirus, and respiratory syncytial virus [128]. It has been possible to study microbial growth, antiviral treatments, and responses to infection by host epithelium‐derived organoids. A recent major application of lung organoids is investigating the SARS‐CoV‐2 virus infection [129]. Most studies are focused on how viruses replicate and affect host cells, especially the expression of receptors angiotensin‐converting enzyme 2 (ACE2) and transmembrane serine protease 2 (TMPRSS2) in different lung cells [130]. Organoids are also being used to identify drugs that could block the entry of the virus into the cells, which is a fundamental mode of action for any therapeutic development [131]. The increasingly widespread use of organoids in research on SARS‐CoV‐2 testifies to their potential in the study of respiratory infections [132]. Much of the recent organoid research targets host response and drug discovery.

Technological advancement has greatly enhanced our understanding of human immune responses to vaccines and pathogens. Peripheral blood sampling is the most common data source in human immunology, but essential immune regulatory functions occur in lymphoid tissues like lymph nodes and the spleen [133]. Animal models give some information but often fail to predict human vaccine response accurately [134]. Understanding human lymphoid tissue and cell interaction is essential to improving vaccine development. Scientists developed an in vitro immune organoid system from human tonsils to achieve this. GC‐like structures with clear B‐cell and T‐cell clusters were observed in the tonsil organoid (TOs) cultures starting about 48 h from the start of culture, proceeding to well‐defined clusters by Days 4–7, especially in live‐attenuated influenza vaccine (LAIV)‐treated cultures. Confocal microscopy revealed light and dark zone patterning of the GCs [135]. Single‐cell RNA‐seq was performed to examine GC‐phenotype B‐cell expression, revealing similar profiles across the time course. Among the overexpressed genes were those engaged in antibody secretion and B‐cell signaling, showing functional responses to antigen‐specific stimulation and adding to the concept that these cultures are organoids. It was found that the LAIV elicited more B‐cell differentiation, higher numbers of hemagglutinin (HA)‐specific B cells and plasmablasts, and a stronger neutralizing antibody response than the inactivated influenza vaccine (IIV) or the wild‐type virus [136]. T‐cell activation was varied by vaccine format and in concordance with responses from previously exposed individuals. LAIV elicited a strong and broadly cross‐reactive antibody response, but most importantly, it induced remarkable levels of NA‐specific antibodies critical to blocking virus release from infected cells. One of the key differences between IIV and LAIV is that LAIV tends to aim at a broader range of antigens. Moreover, there was a strong IFN‐I response after LAIV stimulation; this might explain the increased immune response [137].

Another example of the application of immune cell‐containing organoids in infectious diseases is the use of human TOs recently developed to screen immune responses to the SARS‐CoV‐2 vaccine [136]. The B‐cell–follicular helper T‐cell interaction in germinal centers is of utmost importance in generating immune responses and can be monitored in these organoids [136]. TOs retain tonsil cellular elements and support key B‐cells processes [138]. Following immunization with an influenza vaccine, TOs formed characteristic germinal center structures, while B cells migrated and engaged to regulate the response. TOs were also employed to assess an adenovirus‐based SARS‐CoV‐2 vaccine, demonstrating enhanced CD8^+^ T‐cell activation and antibody production, thereby confirming their utility in measuring personalized vaccine responses [136].

To model inflammatory and pulmonary diseases more efficiently, Seo et al. constructed alveolar organoids that contained macrophages derived from hPSCs [139]. This system enabled the investigation of pulmonary disease pathogenesis and the understanding of the role of innate immune cells in their progression.

The gastric organoids were generated from pluripotent or tissue‐derived stem cells, facilitating the study of infectious diseases. These organoids recapitulate cellular heterogeneity in the stomach, where infections by pathogens such as *Helicobacter pylori* may promote gastritis, ulcers, and malignancies [140]. Studies in gastric organoids have enhanced our understanding of *H. pylori* infection and have unraveled factors that may lead to treatments that could prevent gastric cancer. Gastric organoids also help explain how *H. pylori* colonizes the stomach lining [141, 142].

Intestinal organoids have contributed to the understanding of infectious diseases [143]. These organoids contain various epithelial cell types and recapitulate the functions of the intestine, which is a major portal of entry for many pathogens [144]. They have provided insight into pathogen behavior, immune response, and infection mechanisms. Insights gained into various bacterial and viral infections involving *Salmonella* [145], *E. coli* [146], and *norovirus* [147]. Most recently, intestinal organoids are said to support human norovirus replication for research on infection mechanisms and therapies [148]. So, organoid technology offers great promise for research on gastrointestinal infectious diseases.

Cerebral organoid development has created a new model for studying human brain cells that are important in neurological infection research [149, 150]. Pluripotent stem cells‐derived organoids exhibit features reminiscent of developing brain tissue, including active neural networks and functional synapses [122]. They offer a system to study infections, with notable research on viruses like West Nile and Zika, the latter of which reveals links between infection and microencephaly [151, 152]. Further studies are required on its broader impacts, as shown by COVID‐19, when SARS‐CoV‐2 could invade even brain cells, leading to neuronal death [153].

Current organoid cultures do not have the majority of cells present in the surrounding tissue, which are important for understanding the immune system's interactions with any pathogen [14, 154]. To this end, coculture models combining immune cells with organoids have been developed [155]. More advanced systems, such as body‐on‐a‐chip systems [156], connect several organoid cultures to study the impact of infections on a body rather than on individual tissues alone. Furthermore, through interaction with epithelial biology, the microbiota is essential to disease outcomes and is frequently absent in many organoid studies [157]. Setting up coculture systems containing organoids and microbiota has great potential for advancing knowledge regarding infectious diseases. Such cultures are greatly variable, and standardization is currently lacking. However, ongoing help is coming from advances in single‐cell analysis toward better standardization and insights into the nature of host–pathogen interaction [158, 159].

In vitro models for studying autoimmunity are limited by the inability to grow the affected tissue with its immune environment. Coeliac disease is an autoimmune disease in which gluten‐derived peptides trigger mucosal injury by binding to specific HLA molecules [160]. Researchers have obtained ALI duodenal organoids from endoscopic biopsy samples that keep the epithelial structure and immune cells, including T, B, plasma, NK, and myeloid cells [161]. These organoids showed responses to gluten peptides, leading to epithelial destruction, which could be blocked. Further, it was showed that IL‐7 is essential in this process [161]. However, studies in this field are limited due to the abovementioned limitations.

Some of the most mentioned examples of chronic inflammation include inflammatory bowel diseases (IBDs), such as Crohn's disease (CD), which can affect the small intestine, and ulcerative colitis, which affects the large intestine [162]. Patients with IBD suffer from chronic gut inflammation and have a higher risk of colorectal cancer [163]. The exact causes of IBD are unknown; however, it is considered linked to genetic factors, disturbances in the immune system, and imbalance in gut flora [164]. Because it is a multifactorial disease, treatment for IBD remains difficult. Traditional drugs used in its treatment, including 5‐aminosalicylic acid, glucocorticoids, and immunosuppressants, have a limited sphere of action and often produce side effects [165, 166].

Intestinal organoids—both enteroids and colonoids—are self‐organizing entities of cells recapitulating their in vivo counterparts [167]. These entities represent valuable models for disease studies and drug testing. In a study by Sadman Sakib et al., mouse enteroids and colonoids cultured to acquire defined shapes and characteristics were subjected to inflammation, resulting in a disease phenotype somewhat similar to IBD [168]. Delivering TNF‐α_siRNA through graphene oxide was explored to reduce inflammation in those organoids. PEG and dendrimer‐modified small graphene oxide had the best results in this study. The higher the TNF‐α_siRNA/small graphene oxide ratio, the more effective and less inflammation the results were. Thus, organoids have a great place in the testing of drug delivery systems. In addition to synthetic organoids, tissue‐derived organoids can be used [168].

Intestinal organoids can be isolated from patients with active IBD [168]. Various studies have shown that IBD colonic organoids are different from normal tissues in several aspects: they are smaller in size, with increased cell death, disturbed cell alignment, and lower budding capacity [169, 170]. It expresses low levels of tight junction proteins and altered expressions of other proteins, all of which persist even after the resolution of inflammation [171]. These features can be induced in control organoids after treatment with proinflammatory cytokines. Studies have shown that the organoids, upon chronic exposure to inflammation, acquire stable changes and may represent a model for cancer risk in IBD [172]. PDOs have also been shown to undergo DNA methylation changes associated with treatment outcomes and can be used for drug screening in certain conditions. Overall, these organoids have immense potential in studying the various aspects of IBD [172].

Current treatments for IBD mainly focus on reducing immune responses. However, these treatments often are not effective, indicating that the role of other cell types, such as epithelial and mesenchymal cells, may be very important in ulcerative colitis development [173]. Pre‐existing animal models and epithelial organoids used in research had their shortcomings and failed to reproduce the full complexity of clinical ulcerative colitis [174, 175]. More precisely, most epithelial organoids focused on the epithelium alone, with a failure to consider the contribution of the surrounding microenvironment to colitis. Thus, an induced human ulcerative colitis‐derived organoid (iHUCO), including epithelial and stromal compartments, was developed [176]. This model retains many features of colitic tissue, including epithelial barrier impairment. Currently, intestinal epithelial cells are being cocultured in vitro with mesenchymal and T cells; a couple of challenges may impact the realistic replication of the in vivo cell interactions using such models [176]. Analysis of iHUCOs showed specific cellular changes associated with inflammation, such as abnormal intercellular junctions and early signs of fibrosis. Unique immune populations, mainly B cells, were identified in iHUCOs. Evidence from the model implicated the involvement of CXCL8 and its receptor CXCR1 in the progression of ulcerative colitis. Blockage of CXCR1 by repertaxin, CXCR inhibitor, significantly diminished clinical symptoms of inflammation, thus being a potential therapeutic approach for ulcerative colitis [176].

Chronic inflammation triggers fibrosis, which can progress independently once it begins [177]. However, understanding the mechanisms behind gut fibrosis is still limited, necessitating further exploration of the connections between organ fibrosis and the molecular pathways involved. PDOs from intestinal crypts maintain the tissue's genetic and transcriptomic profiles, making them useful for research.

A study by Laudadio et al. used PDOs from patients with CD, ulcerative colitis, and healthy controls to establish a protocol to induce fibrosis [178]. TNF‐α and TGF‐β1 have also been identified as leading in the inflammatory process and fibrosis during IBD [163]. The PDOs were treated with each cytokine separately and in combination to stimulate inflammation‐driven fibrosis. The results showed that treatment with TNF‐α and TGF‐β1 caused fibrosis in all PDOs, both patient and control, and the response was much stronger than single treatments [178]. The response included phenotypic changes in the organoid structure, with changes in cell types where epithelial cells took on a mesenchymal phenotype. Mesenchymal markers were expressed, and specific fibrotic markers were highly expressed in PDOs. Thus, this proves that CD patients' epithelial cells respond differently to fibrotic stimuli than UC patients. While PDOs do not recapitulate the body's complexity, they give valuable insight into how the intestinal epithelium contributes to fibrosis. Thus, the results hint that IBD‐PDOs facilitate studying the fibrogenesis and developing personalized treatments.

Beyond chronic inflammation, immune cell‐containing organoids may also be applied to investigate hypersensitivity reactions, a field not yet explored but highly promising given the central role of immune cells in these responses.

Therefore, based on the results obtained from using immune cell‐containing organoids in chronic inflammation, these cellular structures can be employed for drug development and the study of disease mechanisms. However, due to the complex interactions of immune cells, particularly those between macrophages and fibroblasts in chronic inflammation, it is crucial to incorporate various types of immune cells in the organoids, including macrophages (especially M2 macrophages) and T cells, along with the parenchymal cells of the tissue of interest and fibroblasts. This platform will enable the investigation of fibrosis in liver, lung and other organs.

Although no studies have yet investigated immune cell‐containing organoids for modeling hypersensitivity reactions, this could represent a potential application given the critical role of immune cells in driving these responses.

## 4 Immune Cell‐Containing Organoid Models of Tissue Damage and Wound Healing

### 4.1 Key Immune Cells in Tissue Damage and Wound Healing

By preventing infections and regulating inflammation, the local immune response plays a crucial role in protecting tissues and minimizing chronic inflammation, which could otherwise lead to tissue damage [179]. Neutrophils are the first immune cells to be recruited to the site of injury, where they contribute to both pathogen clearance and the initial phases of wound repair by releasing proinflammatory cytokines [180]. Around 3 days after injury, neutrophils are typically cleared through phagocytosis by macrophages or fibroblasts [181]. Peripheral blood monocytes, which are persistently recruited to the site of injury, undergo activation and differentiation into macrophages. Macrophages play a central role in wound healing and are also an attractive target for therapy in wound‐healing defects [182]. Defective macrophages account for impaired healing in diabetes and aging, or overhealing in fibrosis [183]. Although factors that promote healing macrophages are known, challenges like macrophage heterogeneity and characterizing their single signals remain [184].

### 4.2 Application of Immune Cells‐Containing Organoids in Modeling Tissue Damage and Wound‐Healing Processes

Due to the composition of organoids, they have been used in some studies for wound healing [185, 186]. These organoids can change the recruitment and function of macrophages and other immune cells, thereby helping in regenerative medicine and wound healing [187]. In a study by Wang et al., iPSC‐derived organoids incorporated into a gelatin‐hydrogel substrate after differentiation and used to treat frostbite‐induced skin damage in a mouse model [135]. This study's results show that using this organoid can heal wounds by improving epidermal cell function and remodeling the ECM by fibroblasts. However, further investigations in this study by performing single‐cell transcriptomics analysis showed that transplanted organoids can change the recruitment and function of immune cells, including macrophages [135]. Further, under the influence of the function of these cells, wound healing occurs with higher efficiency. In macrophages, signaling pathways associated with TLRs and NLRs lead to the production of mediators that affect the functions of fibroblasts and epidermal cells [135]. Another study demonstrated that organoids containing keratinocytes, endothelial cells, and fibroblasts can heal skin wounds in a mouse model. In addition to this organoid's effect on accelerating wound closure, it can contribute to healing skin wounds through neovascularization and local inflammation at the site of skin injury [188]. Inflammation can also reduce the ability of organoids to heal wounds. As shown, organoids derived from the intestines of Crohn's patients have a reduced potential for wound repair when treated with TNF‐a [189].

In some cases, organoids composed of different cells can be transformed into better wound‐healing therapies when exposed to various treatments. In a study by Qian et al., organoids produced from salivary glands were used to investigate their regenerative ability further. The results of this study show that these organoids produce and secrete exosomes that increase the regenerative and wound‐healing ability in a mouse model. The results show that these exosomes can improve wound healing by inducing cell proliferation, angiogenesis, and cell migration [190]. However, detailed studies in this field that use organoids containing immune cells to simulate wound‐healing conditions are scarce, and establishing such techniques is of great importance for investigating the effects of drugs and chemicals on wound healing.

## 5 Construction and Medical Applications of Lymphoid Organoids

The primary lymphoid organs, where immune cells develop, differentiate, and mature, are the thymus and the BM. Mature T and B cells often reside in respond to antigenic stimulation in secondary lymphoid organs, including the lymph nodes, spleen, and mucosa‐associated lymphoid tissue [191]. To fully comprehend these processes and determine the elements that influence cell recruitment, interactions, and functions, versatile in vitro models are required. In terms of microarchitectural complexity and physiological functions, lymphoid organoids possess superior biological attributes compared with conventional culture techniques. In the past decade, we have observed an increase in the variety of in vitro lymphoid organoids that recapitulate the intricate multicellular architecture and functions of the original tissue [27]. Ultimately, this approach will minimize reliance on animal models, enhancing translational relevance while addressing ethical limitations. In this section, we elaborate on the use of organoids for modeling human immunological responses.

### 5.1 Bone Marrow Organoid

The BM promotes and controls blood cell production and immune system function by reacting to the biological needs for blood and immune cell generation under conditions of disease and health. Relationships among hematopoietic cells and microenvironment factors are complex to examine processes in humans. Nevertheless, they are critical for understanding the pathophysiology of blood malignancies and autoimmune illnesses [192]. Recent studies have made significant progress in designing the 3D structure of BM organoids via human iPSCs. This state‐of‐the‐art technology able to accurately simulates various characteristics of human BM, including function and structure. This has allowed us to better understand blood cells production and study blood‐related diseases, and assess new therapeutics. In this approach, iPSCs are transformed into BM cells via particular cytokine mixtures and then incorporated in matrices to support BM architecture and promote vascular formation. The generated organoids recapitulate the BM and mimic the features of human hematopoietic organs as confirmed by single‐cell analysis [193]. The production of the engineered organoids supports primary cells growth from healthy donors or blood cancer patients, providing a suitable platform to study malignant cell interactions [193, 194]. In another study, iPSC‐derived BM organoid was used as a physiologically realistic laboratory model to examine hematopoietic formation and inherent defects of hematopoiesis (like an immunodeficiency). In particular, gene‐edited iPSC‐derived BM imitated features of an inherited BM failure syndrome with resembling characteristics of myelofibrosis and hematological attributes [195]. In summary, the BM organoids offer new insights into blood cell formation and cell interactions in homeostatic and diseased conditions.

### 5.2 Thymus Organoid

The thymus is an important organ of the human immune system, comprising various cells, including thymocytes, stromal cells, and epithelial cells. These cells play an important role in the production of mature T lymphocytes and central tolerance [196]. Various studies have shown that the 3D thymus organoid model is much more successful in simulating immune system mechanisms than conventional laboratory models [197, 198, 199]. For example, researchers succeeded in obtaining thymic organoids using decellularized mouse thymus. This technology can increase the survival and function of thymic epithelial cells and have many of the cellular functions of a complete thymus. The thymic organoid was transplanted into mice without a thymus. Results showed that the precursor cells placed in this organoid began to produce mature T lymphocytes. These mice were subsequently able to reject skin transplants because they had immune mechanisms related to T and B lymphocytes [197]. The human hematopoietic stem cells (HSCs) were also differentiated into mature T lymphocytes in the laboratory. However, the levels of differentiation of mature T cell were limited [200]. Another study produces an artificial human thymic organoid consisting of CD34+/CD3− HSCs and an engineered cell line cultured under air‐fluid conditions. In this system, a high percentage of mature T lymphocytes are produced, which have high cytotoxic function [198]. Thymus organoids have been developed using thymic epithelial cells, iPSC, and human PSC with the ability to promote T‐cell growth, display critical negative selection signals such as the autoimmune regulator protein, and enhance Treg formation. These advancements provide foundations for practical patient‐specific thymic organoid systems, enabling research into human thymus activity, T‐cell generation, and transplant immunology [201, 202].

### 5.3 Lymph Node Organoid

The cortex and para‐cortex of lymph nodes are a critical part of the human immune system. Lymph node contains a highly structured arrangement of different cells like B cells and follicular DCs in follicles and T cell in para‐cortex, which is so evolved to produce appropriate adaptive immune responses to specific antigens [203]. Researchers use lymph node stromal precursors and decellularized ECM scaffolds to create a practical synthetic lymph node organoid [204]. Transplantation of organoids into the site of a removed lymph node, the organoid integrates into the endogenous lymphatic vessels, and effectively restoring lymphatic outflow and circulation [204]. After vaccination, organoid facilitates the activation of antigen‐specific immune responses, adopting the features of natural lymphoid tissues [204]. Likewise, the project focused on organs‐on‐chip and utilized the Matrigel and collagen‐based microfluidic system to promotes the creation of lymph node follicles and germinal centers. They found that administering an influenza vaccine to the lymph node system led to plasma cell development, virus strain‐specific antibody generation, and cytokine release similar to that reported in vaccinated human serum [205]. Therefore, this organoid provides valuable insights into how B cells differentiate and how germinal centers function.

### 5.4 Spleen Organoid

**FIGURE 4:**
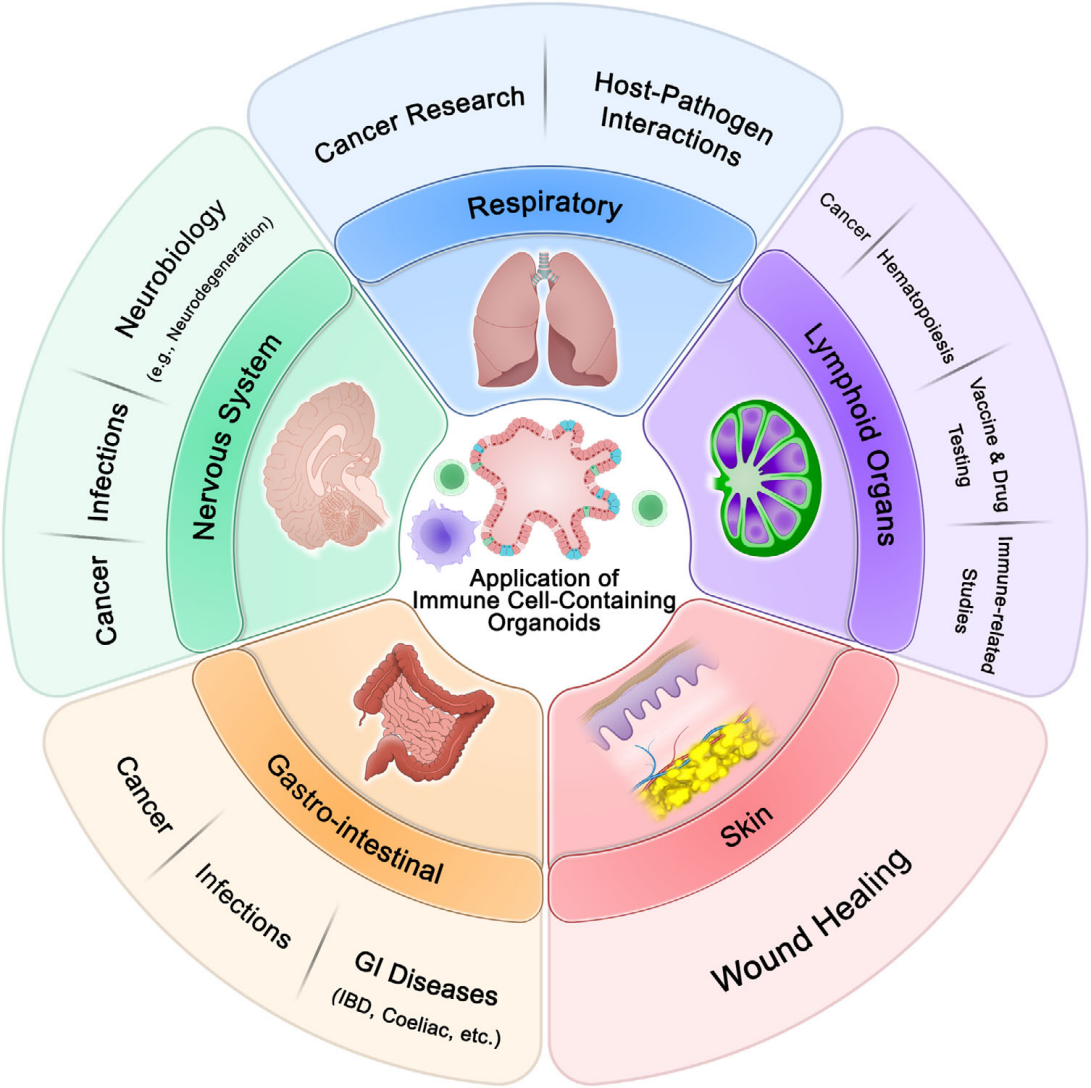
The application of immune cell‐containing organoids in modeling different organs and diseases. The figure illustrates how these organoids replicate pathological conditions of the respiratory, nervous, gastrointestinal, skin, and lymphoid systems.

**FIGURE 5:**
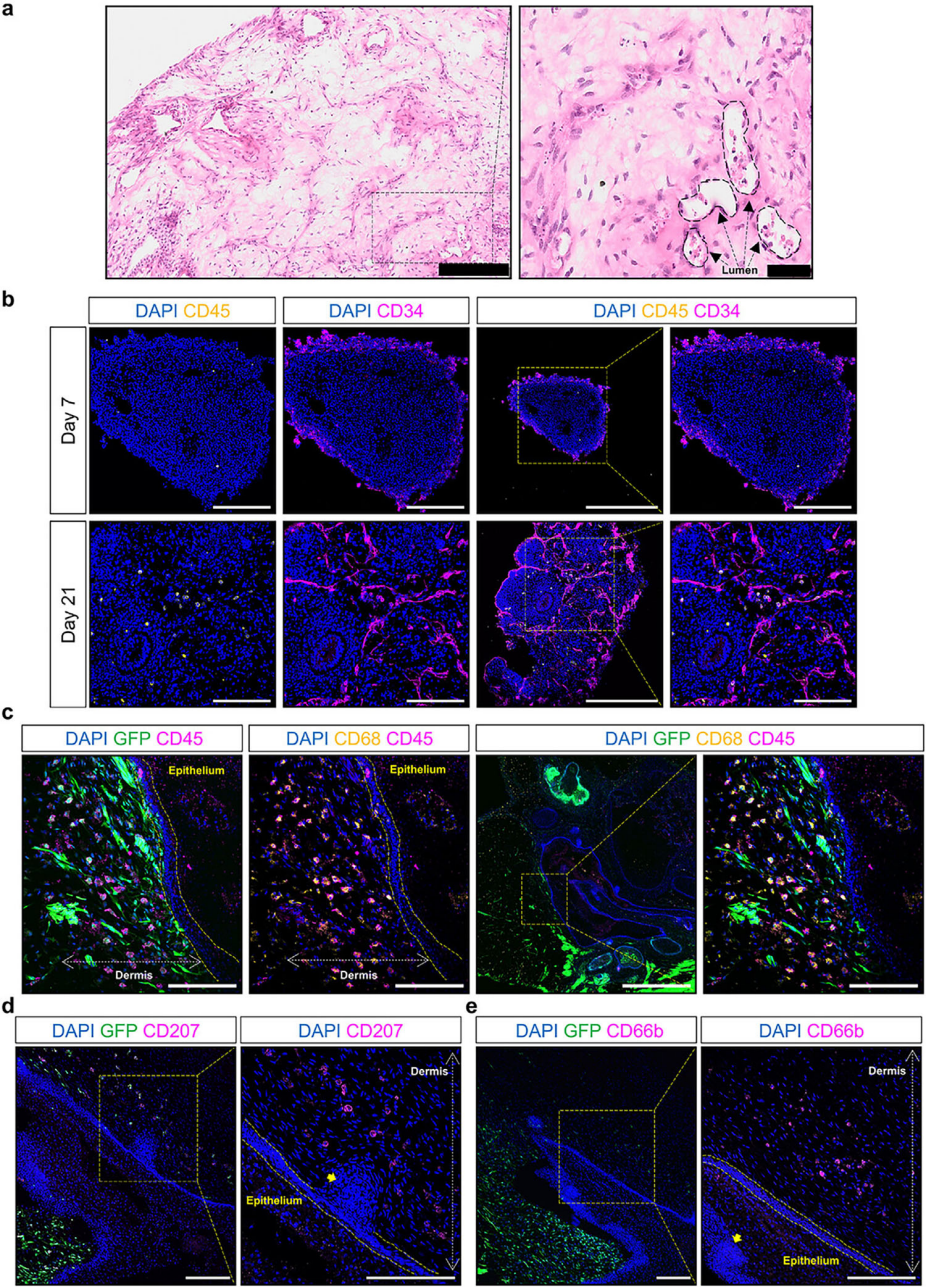
Coordinated development of immune cell‐containing vascularized skin organoids from human‐induced pluripotent stem cells (hiPSCs). (a) Representative hematoxylin and eosin staining images of human‐induced pluripotent stem cell‐derived vascular organoid demonstrate the formation of vascular‐like structures containing immune‐like cells. (b) Immunofluorescence microscopy revealed the emergence of CD45⁺ hematopoietic cells in vascular organoids (VOs) from Day 7 to 21, with some cells coexpressing CD34. Coculture of green fluorescent protein (GFP)‐tagged vascular organoids with GFP− skin organoids (SKOs) was established and confirmed the transfer of CD45⁺ immune cells from vascular organoids into skin organoids. The immune cells were mainly distributed mainly in the dermis part of skin organoids. (c, d) Immunofluorescent staining of vascularized skin organoids on Day 115 of differentiation showed that most of CD45⁺ immune cells were CD68⁺ macrophages, while CD207⁺ cells localized near hair follicles, indicating Langerhans cells or dermal dendritic cells. CD66b⁺ neutrophils were also detected in the dermis, demonstrating the establishment of multiple immune cell types within the vascularized skin organoid. Reproduced from M. Mostina, J. Sun, S. L. Sim, et al. “Coordinated Development of Immune Cell Populations in Vascularized Skin Organoids From Human Induced Pluripotent Stem Cells,” *Advanced Healthcare Materials*, 2025, e02108. https://doi.org/10.1002/adhm.202502108. Figure adapted from open access sources under the terms of the Creative Commons Attribution License (CC BY) [225].

The spleen is the body's biggest secondary lymphoid organ, and serves a variety of immune activities in addition to hematopoiesis and red blood cell/pathogen elimination. The spleen's anatomic architecture facilitates connections among antigen‐presenting cells and lymphocytes [206]. Investigators are using organoid technology to regain the function of human abnormal spleen and examine its immunological properties in the laboratory conditions. An innovation in organoid technology is the use of a porous collagen hydrogel scaffold with specific receptors and stromal cells [207]. When this scaffold is grafted, it causes the filtration of immune cells and their localization in the germinal center of the lymphoid follicle, generating an immune response, but in this model, germinal center events cannot be controlled [207]. In a subsequent study, this problem was solved by using stromal cells expressing CD40 ligand (CD40L) and B‐cell activating factor (BAFF) for induce an accelerated germinal center formation via ECM–cell and cell–cell contact for B cells [208]. In another study, researchers used silicate nanomaterials in the scaffold to improve the efficiency and simulation of organoids, which had high CD40L expression and faster germinal center formation and antibody production [209]. However, as these organoids are of mouse origin, the results cannot be attributed to human biology. Together, all of these studies showed that these organoids have similar structural and functional characteristics to the human spleen. They also showed that the engineered organoids were able to remove red blood cells after transplantation into spleenless mice. Nevertheless, more research is required to fully comprehend the immune reactions in these organoids, particularly regarding T‐cell subtypes and how they play a role in adaptive immunity [210]. The application immune cell‐containing organoids in modeling different organs are shown in Figures 4 and 5. Additionally, immune cell‐containing organoids that mimic different organs are depicted in Table 2.

**TABLE 2:** Development of immune cell‐containing organoids and their application in modeling different organs and diseases.

Organ	Source of organoid	Immune cell	Source of immune cell	Integration method	Purpose	Year of publication	References
Bone marrow	iPSC	Neutrophil, monocyte, eosinophil/basophil/mast cell	iPSC	Differentiation and coculture in Matrigel and Type I and IV collagens	Modeling malignant hematopoiesis in blood cancers	2023	[193]
Bone marrow	BM‐SC from patients	Plasma cell	BM‐SC from patients	Coculture in hydrogel	Modeling blood cancer (multiple myeloma)	2023	[193]
Bone marrow	iPSC	Different subtypes	iPSC	Differentiation and coculture in hydrogel	Modeling physiological functions (hematopoietic formation)	2024	[211]
Bone marrow	Gene‐edited iPSC	Neutrophils, monocytes, macrophages, eosinophil/basophil, mast cells, dendritic cells, lymphoid cells	iPSC	Differentiation and coculture in collagen I/Matrigel matrix	Modeling immunodeficiency	2024	[212]
Thymus	Mouse thymus	T cell	HSC	Differentiation and coculture in decellularized mouse thymus	Modeling physiological functions (production mature T cell and transplant immunology)	2015	[197]
Thymus	Artificial thymus	T cell	HSC	Differentiation and coculture in artificial thymus	Modeling physiological functions (production mature T cell)	2017	[198]
Thymus	iPSC	T cell	iPSC	ALI	Modeling physiological functions (production mature T cell)	2023	[201]
Thymus	Thymic epithelial progenitor cell from mouse thymus	T cell	T‐cell precursors from embryonic thymi	Coculture in Matrigel and ALI	Modeling physiological functions (production mature T cell and transplant immunology)	2024	[213]
Lymph node	Mouse lymph node stromal precursors	T cell, B cell, follicular dendritic cells	Mouse lymph node stromal precursors	Differentiation and coculture in decellularized ECM scaffolds	Vaccine testing	2019	[204]
Lymph node	Human blood cells	T cell, B cell, plasma cell, dendritic cell	Primary human blood B‐ and T‐lymphocytes, autologous monocyte‐derived dendritic cells	Coculture in Matrigel and collagen‐based microfluidic system	Vaccine testing	2021	[205]
Spleen	Mouse spleen‐derived stromal cell	B cell	Mouse spleen‐derived naïve B cell	Coculture in nanocomposite hydrogel	Modeling physiological functions	2015	[208]
Spleen	Mouse spleen‐derived stromal cell	B cell	Mouse spleen‐derived naïve B cell	Coculture in gelatin matrix with silicate nanoparticles	Modeling physiological functions	2017	[209]
Midbrain	HiPSC	T cell	PBMC	Coculture in Matrigel	Modeling neurodegeneration in Parkinson's disease	2025	[214]
Lung	hPSC	Macrophage	Hematopoietic progenitor cells	Coculture in Matrigel	Modeling pulmonary fibrosis and drug testing	2021	[30]
Intestine	LGR5+ stem cell	Macrophage	PBMC	Coculture in Matrigel	Modeling intestinal physiology and pathophysiology	2017	[29]
Lung	Bronchioalveolar stem cells	Macrophage	BAL	Direct injection	Modeling distal lung development, infection, and regeneration	2020	[215]
Lung	hPSCs	Macrophage	hPSCs	Direct injection	Modeling inflammatory lung diseases	2022	[139]
Retina	hiPSC	Macrophage	iPSC	Coculture in Matrigel	Modeling retina and retinal diseases	2023	[216]
Thymus	iPSCs	T cell	iPSC	Decellularized scaffold	Modeling T‐cell development	2022	[217]
Neurons	hiPSCs	Microglia	hiPSCs	Suspension‐based coculture	Investigating the effect of microgravity on CNS cells	2024	[218]
Thymus	Murine bone marrow stromal cell line (MS5)	T cell	HSPCs	Codifferentiation within ALI	Studying T‐cell development	2017	[219]
Human immuno‐organoids	Intestine	Tissue‐resident memory T	Gut	Direct injection	Studying tissue‐resident immune responses	2024	[220]
Intestine	Intestinal stem cells	CD4^+^ T cells	Lamina propria	Coculture	Modeling T‐cell‐intestinal epithelial cell interactions during tissue development and inflammation	2021	[221]
Lung	hiPSC	Innate‐like lymphoid cells (ILCs)	Bone marrow	Coculture	Modeling ILC activity and development in chronic inflammatory diseases	2024	[222]
Tonsil	Tonsil, available lymphoid organs	T cell, B cell, DC	Tonsil	Explant‐derived (already‐resident)	Modeling germinal center influenza vaccine responses	2023	[137]
Tonsil	Tonsil, available lymphoid organs	T cell, B cell, DC	Tonsil	Explant‐derived (already‐resident)	Modeling de novo humoral immune responses	2024	[223]
Tonsil	Tonsil, available lymphoid organs	T cell, B cell, DC	Tonsil	Explant‐derived (already‐resident)	Determining Th1 cells functions in influenza vaccination	2025	[224]
Tonsil	Tonsil, available lymphoid organs	T cell, B cell, DC	Tonsil	Explant‐derived (already‐resident)	Modeling germinal center SARS‐COV‐2 vaccine responses	2021	[136]
Skin	hiPSCs	Hematopoietic cells (CD45+), macrophages (CD68+), Langerhans cells (CD207+), and neutrophils (CD66b+)	hiPSCs	Coculture (vascular organoids [VOs] containing immune cell precursors were cocultured with skin organoids [SKOs]) on ALI	Developing physiologically relevant vascularized human skin organoids	2025	[225]

Abbreviations: ALI, air–liquid interface; BAL, bronchoalveolar lavage; BM‐SC, bone marrow stem cell; CD4+, cluster of differentiation 4 positive; DC, dendritic cell; ECM, extracellular matrix; hiPSCs, human‐induced pluripotent stem cells; hPSC, human pluripotent stem cells; HSC, hematopoietic stem cell; ILCs, innate‐like lymphoid cells; iPSC, induced pluripotent stem cell; LGR5+, leucine‐rich repeat‐containing G protein‐coupled receptor 5 positive; MS5, murine bone marrow stromal cell line; PBMC, peripheral blood mononuclear cell; T cell, T lymphocyte; Th1, T helper 1; TIL, tumor‐infiltrating lymphocyte.

## 6 Sources of Immune Cells for Integration Into Organoids

### 6.1 Direct Isolation From Adult Tissues, Tumor Tissue, or Blood

Isolating immune cells directly from target tissue yields heterogenous cell populations that closely resemble the in vivo counterparts. However, if the target cell population is rare, then obtaining a sufficient number of cells may not be possible. In addition, the isolation process can negatively impact cell viability and the expression of specific cell surface markers [226], necessitating careful consideration in experimental design.

Immune cells could be isolated from blood, tumor tissues, or organ tissues and then integrated into organoids.

Dijkstra et al. cocultured autologous peripheral blood mononuclear cells (PBMCs) with patient‐derived tumor organoids [97]. The coculture of PBMCs with autologous tumor organoids has shown potential in NSCLC for expanding tumor‐reactive CD8+ T cells [97]. These expanded CD8+ T cells demonstrated a significant reduction in the survival of corresponding PDOs, highlighting the utility of this coculture model for assessing immuno‐oncology therapy responses in a personalized manner and for generating patient‐specific tumor‐killing T cells.

Although the isolation of tumor‐reactive T cells and TILs is often fast and well established, this method may be limited by the low number of TILs in poorly infiltrated “cold tumors” [227]. Thus, deriving tumor‐reactive T cells from peripheral blood enables broader applications, particularly when PDOs can be successfully generated from fine‐needle biopsies (FNB) [227]. A recent study developed a coculture method utilizing patient‐derived cholangiocarcinoma (CCA) organoids and immune cells to explore anticancer immunity in vitro [228]. In this study, CCA organoids were cocultured with PBMCs or T cells to assess immune responses. The authors monitored organoid and immune cell behavior. The results showed a significant antitumor response, evidenced by decreased organoid viability, increased apoptosis, and higher cytokeratin 19 fragment (CYFRA) release [228]. Notably, interpatient heterogeneity was observed, and the cytotoxic effects were attributed to both direct cell–cell interactions and the release of soluble factors. This coculture method represents a promising approach for personalized immunotherapy in CCA, offering insights into which immune ICIs may be most effective for individual patients [228].

Overall, while isolating immune cells directly from target tissues provides valuable insights into immune responses, challenges such as limited cell number, cell availability and cell longevity must be carefully considered in generating immune‐integrated tumor organoids.

### 6.2 Immune Cells Differentiated From Induced Pluripotent Stem Cells

Immune cells could be generated from iPSCs. iPSCs‐derived immune cells offer a scalable and patient‐specific source. The iPSC‐derived immune cells are counted as an optimal choice for studying immune responses and disease modeling [229]. To date, various immune cells, including monocytes, DCs, NK cells, T cells, B cells, and neutrophils, have been generated from iPSCs. While there are well‐established protocols for the isolation of immune cells such as monocyte populations from iPSCs, the production of T cells from iPSCs remains challenging due to the complex maturation processes of T‐cell development, necessitating advances to enhance functional maturation [230]. In a recent study, Jurickova et al. successfully integrated macrophages derived from iPSCs into human intestinal organoid (HIO) to investigate an small molecule as a prediction marker for CD [231]. Another study demonstrated that human colonic organoids (HCOs) derived from pluripotent stem cells generate a heterogeneous population of immune cells [232]. These include hemogenic endothelium (HE)‐like cells and erythromyeloid progenitors, which follow a defined differentiation pathway to ultimately produce functional macrophages that can be implicated to model intestinal diseases [232]. Additionally, to enhance cellular diversity and create a more representative model of the retina, human iPSC‐derived macrophage precursor cells (MPCs) were incorporated into retinal organoids containing microglia [216]. To engineer artificial thymic organoids (ATOs) for studying T‐cell development, iPSCs, or hematopoietic stem and progenitor cells (HSPCs) have been used and differentiated into mature T cells [198, 217].

Pirjanian et al. investigated the impact of microgravity on CNS cells by developing neural organoids containing isogenic, iPSC‐derived microglia and sending them to the International Space Station to investigate disease‐specific pathways that may be altered or exacerbated under microgravity conditions [218].

Previous reports have shown that iPSCs are suitable sources for the generation of immune cells, particularly T cells and macrophages, which can be integrated into different organoids. Therefore, further optimization of differentiation protocols is required to generate immune cells in an efficient and scalable manner. Even though the cost and ethical concerns might limit the application of this source, utilizing iPSCs has many advantages, including the capacity to produce desired cell types and their relevance to patient‐specific samples [230].

### 6.3 Immune Cells Differentiated From Hematopoietic Stem/Progenitor Cells of Blood or Bone Marrow

HSCs give rise to all blood cell types including myeloid and lymphoid lineages. Myeloid cells include monocytes, macrophages, neutrophils, basophils, eosinophils, erythrocytes, DCs, and megakaryocytes or platelets. Lymphoid cells include T cells, B cells, and NK cells [233]. HSCs are found in the adult BM, as well as umbilical cord blood, placenta, and mobilized peripheral blood (MPB) [234]. The differentiation of blood [235] or BM‐derived cells [219] provide the benefit of accessibility and the potential for scalability, which are preferable if immune cell‐contained organoids are used for drug screening purposes. In comparison to iPSC‐derived immune cells this approach is more economical [236]. A serum‐free, ATO system has been developed that facilitated the efficient and reproducible in vitro differentiation and positive selection of conventional human T cells from various sources of HSPCs including adult BM, granulocyte colony‐stimulating factor (G‐CSF) MPB, and nonmobilized peripheral blood (PB) [219]. T cells derived from ATOs exhibit mature naïve phenotypes, a diverse TCR repertoire, and TCR‐dependent functionality [219]. In summary, differentiating HSPCs from blood and BM offers a readily accessible, scalable, and cost‐effective strategy for generating diverse immune cell populations.

## 7 Methods of Integrating Immune Cells Into Organoids

Several approaches including coculture models, scaffold systems, direct injection, explant‐derived organoids, and ALI culture offer distinct advantages for integration of immune cells to immune cell‐containing organoids.

### 7.1 Coculture Models

The coculture model is a straightforward and effective method for integrating immune cells into organoids, offering flexibility in including immune cell types. This approach involves growing immune cells alongside organoids and coculturing them, enabling direct interaction between the organoids and immune cells within a controlled environment.

The coculture system can be used to model organs. For example, in a recent study, Gerasimova et al. cocultured T cells isolated from PBMC and human midbrain organoids to understand the role of T cells in neurodegeneration and their interaction with resident cells of the brain in Parkinson's disease (PD) [214]. This approach has also been applied to tumor organoids. The organoid–immune cell coculture system can be utilized to create PDOs models for investigating immunotherapy and interactions between TILs and tumor epithelium. In various cancer types, such as melanoma, breast cancer, pancreatic cancer, and lung cancer, PDO coculture with PBLs or PBMCs has been used as a viable platform to examine personalized immune treatment responses [91, 97, 98, 99].

In PDOs, two approaches exist for establishing coculture systems [237]. The holistic approach retains all cell types, including endogenous immune cells, within the PDOs. In a high‐grade serous ovarian cancer (HGSC), PDO models were utilized to explore the effects of ICIs. The researchers established organoid/immune cell cocultures and performed immune functional assays alongside single‐cell RNA sequencing (RNA‐seq) transcriptional profiling to assess the immune responses. These cocultures were treated with a bispecific anti‐PD‐1/PD‐L1 antibody, in comparison to monospecific anti‐PD‐1 or anti‐PD‐L1 antibodies. This approach enabled the identification of key immune checkpoint blockade targets and revealed significant changes in the cellular states of T and NK cells. These findings underscore the importance of coculture systems in understanding immune cell behavior and therapeutic responses in cancer models [100].

The reconstitution approach entails the individual expansion of PDO and exogenous immune cells prior to their coculturing. This approach involves the expansion and activation of immune cells, facilitating short‐term culture and functional studies. The tumor cells can be isolated through trypsin digestion, followed by coculturing with matched PDOs, both with or without immune cells, to investigate tumor–stromal interactions [89].

Nevertheless, finding suitable exogenous immune cells may be challenging, and these cells may not fully represent the native immune response of the individual in the TME. Future studies could explore longer term cocultures or refine techniques to mimic the native immune environment better, enhancing the relevance of in vitro models for immune response and therapeutics efficacy. As the infiltration and the behavior of the immune cells could significantly alter the TME and tumor progression, it is necessary to conduct more studies on different types of immune cells, especially Tregs, which play an essential role in immune suppression, or other cells like TAMs.

Even though the coculture approach has a wide range of applications, it is important to note that one limitation of this method is the infiltration of immune cells into the inner part of organoids. Additionally, only the cells in the outer layers interact mainly with immune cells, while the cells within the organoid's core are not exposed to immune cells.

### 7.2 Scaffold Systems

Scaffolds and matrices are used to develop 3D models that replicate the structure of native tissues [238]. Scaffold‐based organoids are created by embedding cells into a scaffold, a biomaterial that provides structural support, promotes cell adhesion, and facilitates the organized formation of tissue‐like architectures. Scaffolds are typically made from hydrogels, polymers, or ECM components. Scaffolds offer several advantages, such as creating a supportive microenvironment that encourages cell differentiation and tissue formation, and replicating key features such as cell positioning and ECM composition. Scaffolds also allow the incorporation of specific cell types, including patient‐derived cells or a wide range of immune cells [239], enabling personalized medicine and disease modeling. These structures are valuable tools for studying organ development, physiology, and pathology under controlled laboratory conditions [240, 241]. However, determining the most effective scaffold system remains a key challenge [242]. The scaffold‐based organoid systems face some other limitations: (1) Biocompatibility and structural suitability: the scaffolds must support growth and viability, but may cause suboptimal differentiation or alter cell lineage if incompatible [240, 241]. (2) Reproducibility issues: complex fabrication processes can introduce variability, affecting the consistency of outcomes [243]. (3) Nutrient diffusion limitations: scaffolds can hinder nutrient penetration, leading to hypoxia and growth deficiencies in core areas [244]. (4) Material‐induced changes: scaffold materials may alter cell behavior, differentiation, and signaling pathways [244]. (5) Limited replication of native complexity: scaffolds often fail to fully mimic the intricate cellular organization and relationships found in vivo [245]. In addition, traditional bioscaffold materials often complicate the detachment of tumor organoids and hinder the direct contact between the tumor and cocultured cells. To address these limitations, Shan et al. introduced an innovative acoustic virtual 3D scaffold (AV‐Scaf) method for 3D tumor organoid culture [101]. This approach facilitates a direct‐interacting tumor organoid–immune cell coculture system by leveraging a vortex acoustic field to promote the self‐organization of tumor cells. The AV‐Scaf method enables cell bioassembly and activates ion channels, significantly enhancing calcium ion influx and accelerating intercellular interactions within cellular assemblies. This will be further discussed in the conclusions and future perspectives section.

Moreover, natural and synthetic biomaterials, including collagen and gelatin methacrylate (GelMA), can be engineered to enhance scaffold‐based organoids by providing tunable degradation and stiffness properties, while also supporting differentiation and proliferation, thereby maintaining structural integrity [246]. Since environmental properties such as stiffness can influence immune cell function [247], including TAMs, these platforms may contribute to establishing a more stable environment in tumor immune cells containing organoids.

While scaffolds have been widely used in organoid development, their application in constructing immune cell‐containing organoids is limited due to the challenges mentioned earlier, and the field has shifted toward scaffold‐free organoid systems.

### 7.3 Direct Injection of Immune Cells Into Organoids

Direct injection of immune cells into organoids (lumen) has been proposed to facilitate immune cell integration and ensure effective infiltration [248]. This method provides effective integration, particularly for studying alveolar macrophages. In one study, macrophages from human bronchoalveolar lavage (BAL) were injected into mature bronchioalveolar organoids [215]. These macrophages survived for up to 28 days, performing physiological functions, like [248] surfactant uptake and interacting with alveolar cells via the production of the tight junction protein connexin43 (Cx43) [215]. Another study showed that alveolar organoids with injected macrophages derived from iPSCs responded to lipopolysaccharides (LPS) stimulation by secreting interleukin‐1 beta (IL‐1β) and TNF alpha (TNF‐α) [139]. This integration approach ensured the proper location of macrophages within the alveolar organoid and led to the creation of a multicellular organoid that better mimics human fetal lung [139]. The direct injection method is feasible when precise placement and distribution of immune cells within an organoid are required, but it cannot be applied to all tissues. Because injections are performed into the lumen, this approach is most suitable for lumen‐containing organoids such as intestinal or lung models [248]. In contrast, small, fragile, or densely packed organoids benefit less from this technique, which is also labor‐intensive [249]. Therefore, coculturing organoids with immune cells may provide a more suitable alternative.

### 7.4 Explant‐Derived Organoids (Immune Cells Are Already Present in the Tissue)

The explant tissue samples that contain resident immune cells present a unique advantage by retaining the native microenvironment. In this approach, immune cells are not provided from an external source and fully match the other cells in an organoid. This technique is widely used in cancer research because tumors inherently contain immune cells, serving as a readily accessible source. Moreover, the characteristics of these native immune cells play a critical role in predicting personalized responses to ICB therapy. To date, this technique has been applied across a wide range of tumor organoid models, including melanoma, NSCLC, breast cancer, ovarian cancer, renal cell carcinoma [92], colorectal cancer [94], and CNS tumors [90]. This method has also been used to explore responses of melanoma and CNS tumors to ICIs [90, 96]. These models better replicate in vivo conditions compared to existing in vitro or in vivo models, enabling more precise drug response evaluation and better understanding of tumor–immune interactions and personalized treatments. Explant‐derived organoids often show batch‐to‐batch variability because they are derived directly from patient or tissue samples, which can differ in quality, composition, and growth potential. This makes reproducibility a challenge. Likewise, expanding them in large numbers while maintaining stability and function requires further scalability optimization (e.g., standardized culture conditions, defined media, and automation) since the explants have limited longevity [250, 251].

### 7.5 Air–Liquid Interface

**FIGURE 6:**
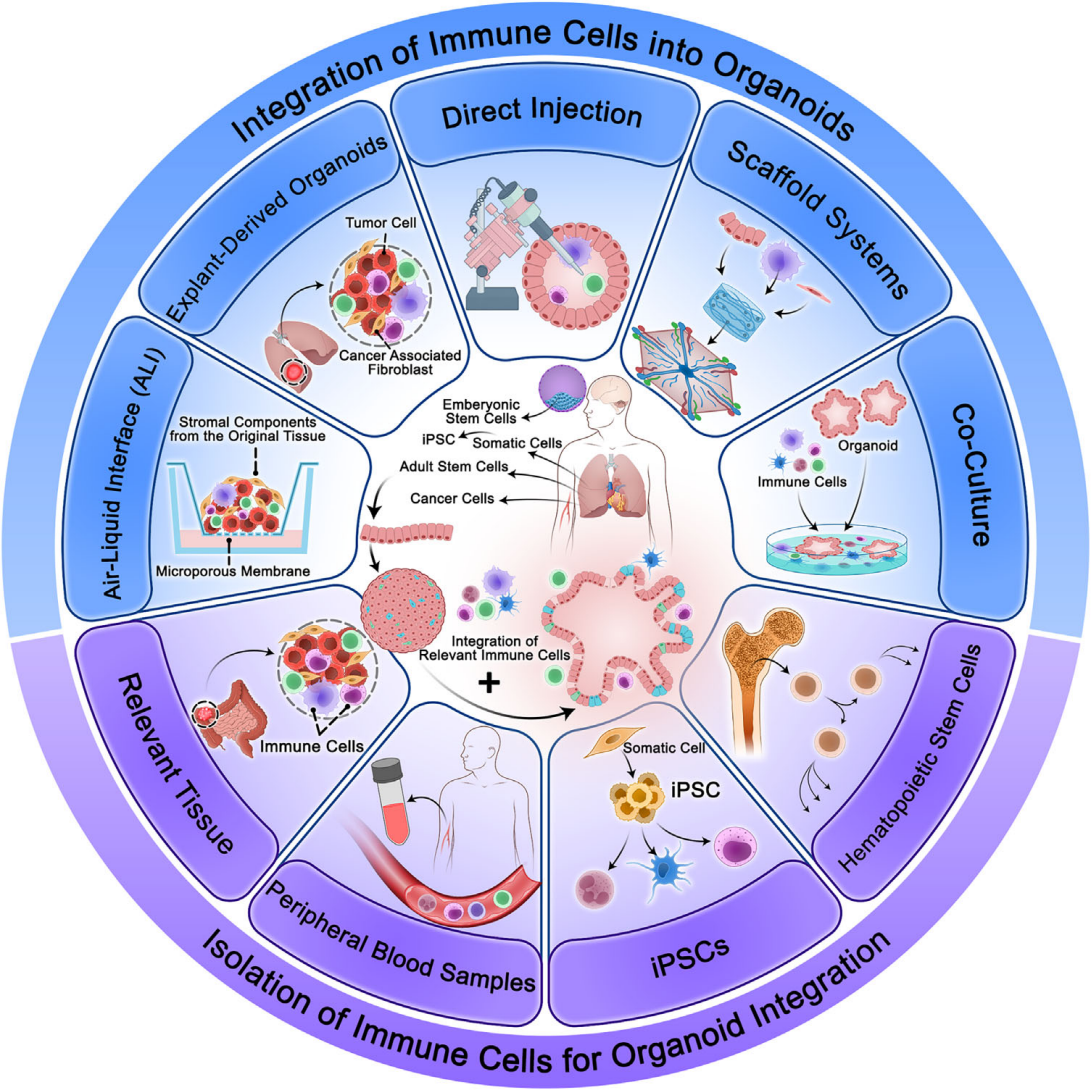
Sources and incorporation methods of immune cells in organoids. The figure shows how immune cells derived from tissue, peripheral blood, iPSCs, and hematopoietic stem cells are integrated into organoid systems using various incorporation techniques. iPSCs, induced pluripotent stem cells.

The ALI system has been widely utilized to enhance the differentiation of various cell and tissue types and has proven effective for culturing diverse organoids, including those of the brain [252], kidney [253], airway [254], and skin [255]. Using ALI, PDOs can grow in collagen gel within a Transwell insert exposed to air, ensuring adequate oxygen supply. The culture medium in the outer dish diffuses into the collagen gel through the permeable Transwell. This system has been used to establish various gastrointestinal cancer organoids by transforming primary organoids from the mouse colon, stomach, and pancreas. Neal et al. extended this method, successfully culturing PDOs from surgically resected primary and metastatic tumors, with a 73% success rate [37]. They introduced an ALI method to culture PDOs that maintain native TILs within the TME. This approach preserves the spatial architecture and cellular composition of the original tumor, enabling the study of endogenous immune responses. Notably, the ALI‐PDOs responded to immune checkpoint blockade therapies, such as anti‐PD‐1 and anti‐PD‐L1, by activating tumor‐specific TILs and inducing tumor cell cytotoxicity [37]. A key advantage of this system is its ability to preserve stromal components from the original tissue, without needing reconstitution, although stromal cells are gradually lost with passaging. In this system, CD4+ and CD8+ TILs were abundant by Day 7 and could be sustained for over a month with IL‐2 supplementation, preserving the intratumoral TCR repertoire. CD11b+ TAMs, along with B cells and NK cells, were also observed in the coculture. This highlights the potential of the ALI method for advancing cancer research and immunotherapy, while also emphasizing the need for further investigation into the long‐term immune cell viability, functional maintenance, and stromal cell dynamics. The sources of immune cells and methods for integrating them into immune cell‐containing organoids are shown in Figure 6.

Although each method presents unique benefits, challenges such as reproducibility, immune cell infiltration, and long‐term stability remain. Continued improvement of these techniques will be essential to establish more reliable immune cell‐containing organoid platforms for different applications.

## 8 Challenges and Limitations of Immune System Organoids and Immune‐Integrated Organ‐Specific Organoids

Although organoids and immune cell‐containing organoids hold great promise as platforms for disease modeling, several challenges, including heterogeneity, structural complexity, difficulties in achieving proper cell maturation and function, and ethical issues, have to be carefully considered.

### 8.1 Heterogeneity

One of the most critical concerns in organoid engineering is overall variation. Organoid cultures may vary significantly in content and functions, resulting in contradictory findings throughout trials. Organoid variation is influenced by beginning factors of development such as the early cell group, culture techniques, and environmental variables. The intricacy of organoid creation techniques, that sometimes need numerous trial stages, particularly for PSC‐derived organoids, is a significant barrier to automation [256, 257].

As a result, procedure standardization is critical to ensuring repeatability and validity in organoid studies. A plural attempt must be undertaken to establish precise rules and methods for assessing the efficacy and reliability of every approach [258]. Single‐cell analyzing methods for transcriptome and epigenome studies can be essential in regard to very precise experiments ideal for this goal [26].

### 8.2 Complexity

Organoid culture is more sophisticated than 2D cell culture. None of the existing organoid technologies replicates the whole function of its individual organ. Organoids usually do not have important particular cell groups, cell–cell interaction, and microenvironment, and unable to reproduce the intricate structure of original organs, due to the lack of mesenchymal cell, endothelial cells, immune cells, blood vessels, and/or microbiota [23, 259, 260]. Coculture techniques including various kinds of cells, and even interorgan communication, are not well‐developed. Although multicomponent organoids have been produced, they do not have uniform cellular structure, making it difficult to conduct reliable and accurate experiments. Unfavorably, a consistent methodology or instructions covering these concerns are still absent, therefore each scientists are forced to find the most suitable approach for themselves [261].

### 8.3 Low Level of Maturity and Function

When organoids expand in size, they face challenges in nutrition delivery and waste disposal. Bigger organoids can develop necrotic areas because of inadequate nutrition transport, complicating study results. In spite of nutrition availability issues, inaccessibility complicates management of an organoid's many compartments. Studies using epithelial organoids, like gut organoids, usually need availability to the epithelium's both inside and outside surfaces. The use of flow, a gas interface or mechanical stimulation may increase ultimate maturity of cells [262, 263]; despite that, integrating such characteristics stays technically difficult. As a result, organoids' short lifetimes are frequently the direct result of limited availability. This brief period limits the capacity to examine long‐term impacts or completely mature the cells toward adult characteristics. This culture period constraint is much more troublesome in PSC‐derived organoids, that lifetimes are vastly different from the duration of in vivo organ development, particularly in human systems [15, 264].

### 8.4 Ethical Concerns

In spite of their scientific potential, organoids are not considered ethically neutral. The ethical issues of organoid technology are likewise challenging. Organoid systems generate multiple important ethical concerns involving the origin of stem cells for their production, informed consent and privacy of cell donors, the moral and lawful condition, the application of modification of genes, generation of chimeras, organoid transplantation, marketing and licensing, concerns about equality of the resulting therapies, and lasting storage in biobanks. In particular, donors of cells might find long‐term personal links with their organoids, raising the threshold for informed consent and the individual engagement. In addition, certain organoid categories, like neuronal organoids and human–animal chimeric organoids, have sparked debate [265]. In spite of the substantial ethical issues raised by organoid application and biological banking, we have a moral commitment to encourage organoid investigations and guarantee that we never forfeit any of the significant advantages that organoids provide [266].

In summary, organoids show enormous potential for improving the comprehension of human physiological processes and illness, but overcoming these obstacles is critical to their successful use in science. The creation of organoid technologies should be reconsidered to enhance the range of adjustable factors. Engineering techniques, commonly investigated by proof‐of‐concept research on organ‐on‐a‐chip technology, provide the prospect of addressing numerous shortcomings of organoid systems [267].

## 9 Future Perspectives

### 9.1 Bioengineered Organoids

To create a more controlled environment that mimics the native tissue characteristics including chemical gradient, microfluidics are more applicable [268]. This technology offers the benefits of quantitating the cell interactions, investigating cell migration more precisely [269] and integrating sensors [270]. For instance, Wu et al. studied the relationship between neutrophil recruitment and migration of endothelial cells via a microfluidic model [269]. The immune cell‐containing microfluidic organoids could be created in the future to investigate the migration and metastasis of cancer cells in cancer organoids, or to study the process of wound healing in skin organoids [22]. More innovatively, microfluidic chips could be combined with 3D bioprinting, which is an advancement to achieve accurate structure and arrangement of organoids [271]. This combination has been used to study the TME dynamic flow and biostructure [271]. This technology alone has been used to integrate macrophages into brain glioblastoma organoids [272].

In addition, organoids could also be fused with organs‐on‐chips technology, creating organoids‐on‐a‐chip [273, 274, 275]. Ogans‐on‐chips are controllable artificially constructed organs that could be sophisticated models to advance cancer immunotherapy as comprehensively reviewed by Zhu et al. [276]. Overall, immune cell‐containing organoids, enhanced by microfluidic technology, 3D bioprinting, and organ‐on‐a‐chip systems, hold great promise for advancing biomedical research. The integration of bioengineering strategies, such as microwell arrays, bioreactors, microfluidics, bioprinting, and hydrogel matrices, enhances organoid maturation, scalability, and functionality paving the way for clinical translation despite the current mentioned challenges, like vascularization, heterogeneity, and ethical issues [277]. These platforms enable precise control over cellular interactions, migration, and microenvironmental factors, making them valuable tools for studying cancer metastasis, immune responses, and tissue regeneration.

### 9.2 Biobanking of Organoids

Long‐lasting culture, growth, and preservation of organoids provide the required conditions for the construction of biobanks, which standardize the collecting and conservation of healthy or patient‐derived samples, along with relevant medical data [278]. Most organoid research institutions create an organoid biobank using healthy and diseased tissue, stem cells and organoids and these specimens are exchanged among organoid research centers. These centers share experiences and approaches and offer a comprehensive and consistent platform for translational studies. These resources are typically accessible to scientists from the host school or organization. However, some institutions make their organoid lines accessible for commercial purchase through ATCC (e.g., Wellcome Sanger Institute, UK) or straight from them [279].

The importance of immune cell coculture assessments in personalized clinical studies necessitates the development of specialized biobanks that preserve and sustain both organoid and blood specimens from the same individual (e.g., the Organoids in 3D condition, Istituto Nazionale di Genetica Molecolare, Italy; the Human Organoid Facility & Biobank, Institute of Cancer Research, UK). These biobanks can be particularly useful in studying the interaction of immune cells with cancers or a variety of other cells, such as intestinal epithelial cells, in order to reveal variations in treatment efficacy in malignancy or IBD patients. Donor‐matched specimen collections in biobanks can enable numerous translational study projects, boosting the robustness of the outcomes produced for personalized medicine [280].

The long‐term cultivation of organoids, along with the absence of effective cryopreservation technologies, restricts their subsequent use. Despite effective cryopreservation for small amount of biological specimens such as cells and embryos having been achieved, the current cryopreservation technologies for organoids continue to confront major obstacles [281]. Expensive long‐term organoid cultivation has limited its widespread efficiency. Therefore, the establishment of improved strategies for organoid cryopreservation is essential. Furthermore, the potential effects of prolonged preservation and freeze–thaw cycles as important features of biobanking require further investigation [282]. In the future, overcoming the homogeneity of organoid technical guidelines and establishing high quality monitoring systems are critical to the development of a safe, precise, and accessible biobank, which must receive significant focus from scientists [278].

### 9.3 Artificial Intelligence

AI has significantly advanced organoid research by enhancing image analysis, optimizing differentiation protocols, and enabling large‐scale data interpretation. Deep learning techniques, particularly convolutional neural networks (CNNs), have been applied for automated classification of organoid morphology, cell populations, and drug response profiling [283, 284, 285, 286, 287, 288, 289]. Additionally, CNNs have been employed to predict retinal differentiation in retinal organoids, demonstrating AI's potential for early‐stage organoid assessment [289].

AI has also been integrated into drug discovery workflows, where machine learning models analyze high‐throughput screening data to predict organoid responses to therapeutic compounds [290, 291, 292].

Beyond data analysis, AI is also playing a crucial role in optimizing organoid construction. Machine learning models can be used to refine the composition of ECM hydrogels, ensuring optimal physicochemical properties for organoid growth and immune cell viability. Additionally, AI‐driven automation can enhance quality control by analyzing microscopy images to detect structural abnormalities in organoids. AI also enables real‐time monitoring of culture conditions, adjusting parameters dynamically to maintain stable and reproducible organoid systems [293].

AI also has the potential to revolutionize personalized medicine through patient‐specific organoid modeling. By analyzing how PDOs respond to different treatments and environmental stimuli, AI can help predict individualized treatment responses. Machine learning algorithms can integrate patient‐specific genetic data, disease characteristics, and response patterns to identify the most effective therapies while minimizing side effects. This personalized approach enhances precision medicine by tailoring treatments to each patient's unique biology [293].

An example of AI in disease modeling is its application in neurotoxicity prediction using human midbrain organoids [286]. AI‐based machine learning models have been used to analyze midbrain organoids derived from PD patients, facilitating image‐based cellular profiling. These models can quantify dopaminergic neuron count, neuronal complexity, and cellular responses to neurotoxic compounds.

Additionally, AI enhances data processing strategies, enabling the differentiation between various treatment conditions, which is crucial for drug screening and disease modeling. The effectiveness of this approach has been validated using high‐content imaging data from PD patient‐derived midbrain organoids, showcasing AI's potential in advancing neurodegenerative disease research [286].

Overall, while AI presents transformative opportunities for organoid research, overcoming its current limitations through improved data quality, validation frameworks, and standardized methodologies will be essential to fully harness its potential in biomedical applications. More specifically, machine learning methods have been used to determine the optimal time point for organoid subculture and maintenance [294], quantify organoid morphological features [295], and estimate the patterns of gene‐expression patterns [295]. Hence, machine learning could be applied to determine the location of immune cells within immune cell‐containing organoids, thereby revealing their spatial organization and predicting the outcome of cell interactions and the fate of immune cells.

Despite its opportunities, the integration of AI in organoid research faces several challenges. For example, for PDOs, the patient's data must be strictly protected to ensure privacy and security. In addition, the vast heterogeneity in culture conditions across organoid systems complicate AI model training which can be both resource‐intensive and time‐consuming. Overcoming these technological and ethical hurdles will require the establishment of standardized methodologies, the design of interpretable algorithms, collaborative data‐sharing frameworks, and strong regulatory guidance [296].

## 10 Preclinical Prospect of Immune Cell‐Containing Organoid Transplantation

In addition to their established roles in precision medicine, disease modeling, and drug screening, organoids present promising potential for transplantation into animals or humans to stimulate tissue regeneration and support immune function or improve tissue healing. The ability to provide a high number of diverse cells in a native‐like structure, along with their high survival rate, renders them a promising candidate for transplantation to tackle the challenge of donor organ shortages [297, 298].

For instance, epithelial organoids have been successfully infused into the intestinal lumen of mouse models with colonic injury. After their transplantation, epithelial organoids localized to the site of injury and regenerated donor‐derived epithelium [299]. Although this approach holds potential for treating colonic injury in conditions such as ulcerative colitis and other forms of IBD, the donor origin of regenerated cells may initiate host immune responses, leading to organoid rejection, which remains to be an ongoing challenge in organoid transplantation.

Beyond the gastrointestinal system, the therapeutic application of organoid transplantation has also been explored in other organs. Cerebral organoid transplantation in animal models of stroke and traumatic brain injury has demonstrated therapeutic benefits, including supporting neural growth, maturation, and integration into host circuits, enabling their use in therapy, cell studies, and disease modeling [297]. In addition, advances in organoid transplantation offer a potential alternative for end‐stage liver disease, addressing the critical shortage of donor livers. Liver organoids can both treat liver diseases and repair liver grafts ex vivo, thereby enhancing the supply of transplantable liver tissue [298].

Despite their promise, organoid transplantation could be limited by immune rejection. In kidney organoids, coculture of iPSC‐derived kidney organoids with PBMCs led to immune cell invasion and fibrosis, while in vivo implantation resulted in infiltration of diverse T‐cell subsets. These findings demonstrate that organoids, like conventional transplants, are vulnerable to allo‐immune attack, highlighting immune compatibility as a major challenge for clinical use [300]. These obstacles could be overcome by using autologous sources for both organoids and immune cells, thereby minimizing the risk of rejection [297, 301].

Currently, no clinical trials investigating the transplantation of immune cell‐containing organoids in humans have been reported. Patients with acute or chronic organ injuries, such as ulcerative colitis, hepatitis, stroke, or burns and scars on the skin, could potentially benefit from these therapies. Incorporating immune cells into organoids may help modulate host responses, promoting a balanced and beneficial immune environment, rather than a suppressed or dysregulated one.

## 11 Conclusions

In conclusion, immune cell‐containing organoids represent a transformative approach in biomedical research, offering new possibilities for more precise and physiologically relevant investigation of personalized cancer treatments, infection, inflammation, autoimmunity, regenerative medicine, and primary lymphoid organs. Their continued refinement and integration with cutting‐edge technologies will pave the way for more effective and individualized therapeutic interventions and studies, with the potential to refine our understanding of human immunology, disease mechanisms, and therapeutic strategies. By addressing current limitations such as cell and organoid heterogeneity, complexity, low maturation levels, and ethical concerns, the next generation of organoid models is able to drive breakthroughs in personalized medicine, immuno‐oncology, and regenerative therapies.

## Author Contributions

Conceptualization: Abbas Shafiee and Seyed Mahmoud Hashemi. Investigation: Abbas Shafiee, Seyed Mahmoud Hashemi, and Kimiya Rashidan. Visualization: Amirhossein Nazerian. Supervision: Abbas Shafiee and Seyed Mahmoud Hashemi. Original draft preparation: Kimiya Rashidan, Malaksima Ayadilord, Ali Hazrati, and Amirhossein Nazerian. Review and editing: Kimiya Rashidan, Seyed Mahmoud Hashemi, and Abbas Shafiee. All the authors have read and approved the manuscript.

## Funding

The authors received no specific funding for this work.

## Ethics Statement

The authors have nothing to report.

## Conflicts of Interest

The authors declare no conflicts of interest.

## Data Availability

All data supporting this study are included within the article.
